# Fission Yeast Scm3: A CENP-A Receptor Required for Integrity of Subkinetochore Chromatin

**DOI:** 10.1016/j.molcel.2009.01.019

**Published:** 2009-02-13

**Authors:** Alison L. Pidoux, Eun Shik Choi, Johanna K.R. Abbott, Xingkun Liu, Alexander Kagansky, Araceli G. Castillo, Georgina L. Hamilton, William Richardson, Juri Rappsilber, Xiangwei He, Robin C. Allshire

**Affiliations:** 1 Wellcome Trust Centre for Cell Biology and Institute of Cell Biology, School of Biological Sciences, The University of Edinburgh, 6.34 Swann Building, Edinburgh EH9 3JR, Scotland, UK; 2 Department of Molecular and Human Genetics, Baylor College of Medicine, Houston, TX 77030, USA; 3 MRC Human Genetics Unit, Crewe Road, Edinburgh EH4 2XU, Scotland, UK

**Keywords:** DNA, PROTEINS

## Abstract

The mechanisms ensuring specific incorporation of CENP-A at centromeres are poorly understood. Mis16 and Mis18 are required for CENP-A localization at centromeres and form a complex that is conserved from fission yeast to human. Fission yeast *sim1* mutants that alleviate kinetochore domain silencing are defective in Scm3^Sp^, the ortholog of budding yeast Scm3^Sc^. Scm3^Sp^ depends on Mis16/18 for its centromere localization and like them is recruited to centromeres in late anaphase. Importantly, Scm3^Sp^ coaffinity purifies with CENP-A^Cnp1^ and associates with CENP-A^Cnp1^ in vitro, yet localizes independently of intact CENP-A^Cnp1^ chromatin and is differentially released from chromatin. While Scm3^Sc^ has been proposed to form a unique hexameric nucleosome with CENP-A^Cse4^ and histone H4 at budding yeast point centromeres, we favor a model in which Scm3^Sp^ acts as a CENP-A^Cnp1^ receptor/assembly factor, cooperating with Mis16 and Mis18 to receive CENP-A^Cnp1^ from the Sim3 escort and mediate assembly of CENP-A^Cnp1^ into subkinetochore chromatin.

## Introduction

Kinetochores in most eukaryotes are located in a specific chromosomal region, the centromere, and coordinate accurate chromosome segregation. The incorporation of the histone H3 variant CENP-A (also known as CenH3, *Drosophila* CID, *C. elegans* HCP-3, *S. cerevisiae* Cse4, and *S. pombe* Cnp1) in place of normal H3 is critical for the kinetochore assembly at centromeres. This unusual subkinetochore chromatin is assembled only at active centromeres (reviewed in Cleveland et al., 2003).

Although CENP-A and kinetochores tend to be associated with specific sequences residing at centromeres, it is generally accepted that CENP-A chromatin assembly is epigenetically regulated (Karpen and Allshire, 1997; Sullivan et al., 2001; Sullivan, 2001). Most compelling is the finding that kinetochore proteins can assemble on noncentromeric DNA and form neocentromeres at novel sites spontaneously or when CENP-A levels are artificially elevated. Once established at these new sites, mechanisms must exist to recognize this CENP-A and allow it to be renewed and propagated during cell division. In addition, nascent CENP-A must normally be directed to sites of existing CENP-A chromatin for assembly into chromatin and be prevented from assembling into non-centromeric loci.

Pulse-chase experiments in human cells indicate that CENP-A is incorporated at centromeres in telophase-G1 so that new CENP-A is deposited following centromere segregation (Jansen et al., 2007). Consistent with this, during the rapid divisions in *Drosophila* embryos, new CENP-A accumulates at centromeres in anaphase (Schuh et al., 2007). It seems likely that canonical histone H3 is first deposited during S phase and subsequently replaced or that nucleosomal gaps are created and then filled (Furuyama et al., 2006; Shelby et al., 2000; Sullivan and Karpen, 2001). However, little is known about the components that direct assembly of new CENP-A at centromeres in telophase-G1. In fission yeast, CENP-A is incorporated at centromeres during S phase and G2 (Dunleavy et al., 2007; Takahashi et al., 2005; Takayama et al., 2008); however, proteins required for CENP-A incorporation associate with centromeres in late anaphase but are released in early mitosis (Fujita et al., 2007). Thus, anaphase/telophase appears to be a key point in the cell cycle for regulating and permitting CENP-A deposition.

The histone-binding protein RbAp46/48 is known to participate in a number of histone transactions and has been reported to copurify with *Drosophila* CENP-A and promote CENP-A chromatin assembly in vitro. In fission yeast cells the RpAp48 protein (Mis16) is concentrated at centromeres but dissociates briefly in early mitosis and reappears in anaphase (Furuyama et al., 2006; Hayashi et al., 2004). The RbAp46/48 histone-binding proteins associate with the Mis18 complex, which is also involved in CENP-A deposition. The human complex consists of Mis18α, Mis18β, and Mis18BP1 (also known as KNL2 [Maddox et al., 2007]); all three proteins accumulate at human centromeres in a codependent manner between telophase and G1 and are required for the deposition of newly synthesized CENP-A. Fission yeast Mis16 and Mis18 physically interact and depend on each other for their localization at centromeres (Hayashi et al., 2004). Like Mis16, Mis18 transiently leaves centromeres from early mitosis until anaphase, when it again localizes to centromeres.

It has been proposed that Mis18 and associated proteins may prime the centromere (following the successful completion of metaphase/anaphase) and thus permit the incorporation of CENP-A in subsequent cell-cycle stages (Fujita et al., 2007; Jansen et al., 2007; Maddox et al., 2007). However, although RbAp46/48 associate with *Drosophila* CENP-A, no association of CENP-A with Mis16/RbAp46/48 or Mis18 has been reported in other systems. Thus, the connection between the Mis18 complex and CENP-A incorporation remains unexplained.

Critical residues in the histone fold domain of CENP-A differ from canonical H3 and define the CATD domain required to target CENP-A to centromeres (Black et al., 2007; Sullivan et al., 1994). Comparative affinity purification of CENP-A versus H3.3 mononucleosomes has allowed the identification of proteins that specifically associate with CENP-A nucleosomes including both subunits of FACT, a histone chaperone involved in nucleosome disassembly and reassembly (Foltz et al., 2006; Obuse et al., 2004b). In addition, the CENP-A-nucleosome-associated complex and more distal components have been identified (Foltz et al., 2006; Obuse et al., 2004a; Okada et al., 2006). However, the role of these proteins in CENP-A deposition has not been explored in detail. Thus, although many CENP-A-interacting proteins are known, our knowledge of the specific chaperones required to mediate the CENP-A chromatin assembly is surprisingly sparse.

We previously identified Sim3 in fission yeast, a homolog of the histone-binding protein NASP/N1-N2, which associates with CENP-A and is required for its efficient deposition at centromeres (Dunleavy et al., 2007). However, Sim3 is distributed throughout the nucleoplasm, suggesting that its role is to escort CENP-A to centromeres and hand it over to centromere-associated incorporation factors. The Mis18 complex is clearly a good candidate for this function, but a specific centromere-associated receptor for CENP-A has not been identified in organisms with regional, epigenetically regulated centromeres.

*S. cerevisiae* centromeres are streamlined so that 125 bp of DNA is wrapped around a single CENP-A^Cse4^ nucleosome and is sufficient to specify centromere function. In contrast, in fission yeast CENP-A^Cnp1^ associates with a central domain of ∼12 kb flanked by heterochromatin on both sides (Pidoux and Allshire, 2004). In *S. cerevisiae* the Scm3^Sc^ protein is required for centromere association of CENP-A^Cse4^ (Camahort et al., 2007; Mizuguchi et al., 2007; Stoler et al., 2007) and has been proposed to form part of an unusual centromeric nucleosome with Scm3, perhaps forming a stable hexameric complex with a Cse4-H4 tetramer (Mizuguchi et al., 2007). It is not known if such Scm3 nucleosomes, devoid of H2A-H2B, are a general feature of centromeric chromatin in other organisms. *S. cerevisiae* Scm3 associates with Ndc10, a component of the CBF3 DNA-binding complex that binds the 15 bp CDEIII centromere element and is absolutely required to specify centromere function. Thus, it is possible that the function of Scm3, its connections with CENP-A^Cse4^, and the nature of the CENP-A nucleosomes differs between organisms.

Here we identify and characterize the Scm3 protein of fission yeast. Our analyses indicate that *S. pombe* Scm3 (Scm3^Sp^) is a centromere-associated protein that binds CENP-A^Cnp1^ and is required to maintain the integrity of CENP-A^Cnp1^ chromatin. Scm3^Sp^ is released from chromatin independently of CENP-A^Cnp1^. Furthermore Scm3^Sp^, like Mis16 and Mis18, dissociates from centromeres between early mitosis and anaphase. Reciprocal to this, Scm3^Sp^ remains associated with centromeres in CENP-A^Cnp1^ mutants, where CENP-A^Cnp1^ is lost from centromeres and replaced with H3. Thus, Scm3^Sp^ is unlikely to be an integral component of CENP-A^Cnp1^ nucleosomes, since its association with chromatin is uncoupled from CENP-A^Cnp1^. We propose that Scm3^Sp^ acts to receive CENP-A^Cnp1^ at centromeres and, in cooperation with Mis16 and Mis18, mediates its incorporation into nucleosomes in place of H3.

## Results

### *sim1* Mutants Are Defective in Kinetochore Integrity Due to Lesions in the Gene Encoding the *S. pombe* Scm3 Ortholog

**Figure 1:**
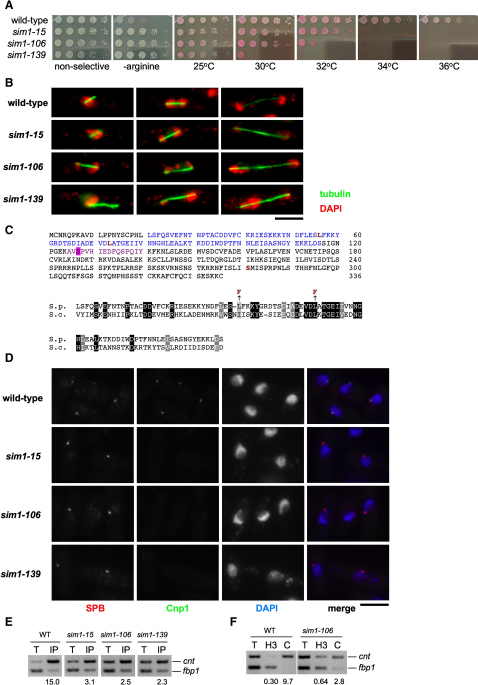
*sim1* Mutants Are Defective in Centromeric Silencing, Chromosome Segregation, and CENP-A^Cnp1^ Association with the Centromere (A) Five-fold dilutions of the indicated strains were spotted on nonselective or medium lacking arginine to assay for alleviation of silencing at the central core (*cnt1:arg3*^+^). To assess temperature sensitivity, strains were spotted onto YES + phloxine B at the indicated temperatures; dead cells stain dark pink. (B) Chromosome segregation defects in *sim1* mutants. Cells were shifted to the restrictive temperature of 36°C for 6 hr, before fixation and processing for immunofluorescence with anti-tubulin (TAT1) antibodies (green) and DAPI staining (red). Spindle length indicates that cells on left in wild-type and *sim1-15* are in metaphase, and all other cells are in anaphase. Scale bar, 5 μm. (C) *S. pombe* Sim1 is similar to *S. cerevisiae* Scm3. *S. pombe* Sim1/Scm3 (SPAPB1A10.02) is predicted to be 37.6 kDa. Blue: equivalent to region of Scm3^Sc^ that binds CENP-A^Cse4^ (Mizuguchi et al., 2007). Red: amino acid changes in *sim1-139* (L56F), *sim1-106* (L73F), and *sim1-15* (S281L). Violet: phosphorylated tryptic peptide detected by LC MS/MS at S127 (Figure S12). Bottom: Alignment of conserved region within the CENP-A^Cse4^ binding domain of *S. cerevisiae* Scm3^Sc^ (aa 74–169) with residues 21–116 of *S. pombe* Scm3^Sp^ using Clustal W*/*BoxShade. Amino acid changes in *sim1-139* (L56F) and *sim1-106* (L73F) are indicated. (D) CENP-A^Cnp1^ localization in *scm3* mutants. Cells were shifted to 36°C for 6 hr, before fixation and processing for immunofluorescence with Sad1 antibodies to decorate spindle pole bodies (SPB; red) and provide a marker for approximate position of centromeres, labeled with anti*-*Cnp1/CENP-A^Cnp1^ antisera (Cnp1; green). DAPI (blue). Scale bar, 5 μm. (E) ChIP for CENP-A^Cnp1^ in the indicated strains, incubated at 36°C for 6 hr prior to fixation. Primer pairs specific for central core (*cnt*) and a euchromatic control locus (*fbp1*) were used in multiplex PCR and used to calculate enrichment in IP relative to total input chromatin (T). (F) ChIP for histone H3 (H3) and CENP-A^Cnp1^ (C) and in wild-type and *sim1-106*. Primers and quantification as in (E).

Fission yeast has three centromeres of 40, 65, and 110 kb, each composed of two distinct chromatin domains (reviewed in Pidoux and Allshire, 2004). The outer repeats are coated in heterochromatin, whereas nucleosomes composed of the centromere-specific histone H3 variant CENP-A (CENP-A^Cnp1^: Cnp1 in *S. pombe*) occupy the central domain chromatin and comprise subkinetochore chromatin (Partridge et al., 2000; Takahashi et al., 2000). Transcriptional silencing is imposed on marker genes inserted within either domain (Allshire et al., 1994, 1995). Wild-type strains harboring the *arg3*^+^ gene in the central core (*cnt1:arg3*^+^) of centromere 1 grow slowly on plates without arginine (Figure 1A), indicating that the *arg3*^+^ gene is silenced. Mutants that alleviate silencing of marker genes inserted into the central core region have facilitated identification of kinetochore proteins and factors required for assembly of subkinetochore chromatin (Dunleavy et al., 2007; Pidoux et al., 2003). The three *sim1* alleles isolated in this screen specifically alleviate (Pidoux et al., 2003; data not shown) silencing within the central domain subkinetochore chromatin. *sim1* mutants are temperature sensitive for growth (Figure 1A). As in other mutants affecting kinetochore integrity, the smeared micrococcal nuclease digestion pattern of the central domain is disrupted in *sim1* mutants (see Figure S1 available online). Cytological analyses of *sim1* mutants indicate that they have dramatic chromosome segregation defects such as chromosome nondisjunction, aberrant anaphase with lagging chromosomes, and overcondensed chromatin (Figures 1B and S2). The *sim1*^+^ gene was identified by complementation of the *sim1* mutant phenotypes with a plasmid-borne genomic library. Complementing plasmids contained the ORF SPAPB1A10.02 and sequencing from genomic DNA of the PCR-amplified ORF identified S281L, L73F, and L56F missense mutations in *sim1-15*, *sim1-106*, and *sim1-139*, respectively. Database searches and alignments revealed that the Sim1 protein shares similarity with *S. cerevisiae* Scm3 and related fungal proteins as reported (Aravind et al., 2007). The L73F and L56F mutations lie in conserved residues within the region of Scm3^Sc^ that binds CENP-A^Cse4^ (Figure 1C). Deletion of the gene encoding this Scm3-like protein (henceforth referred to as the *scm3*^+^ gene; *scm3-15*, *scm3-106*, and *scm3-139* alleles; and Scm3^Sp^ protein) in diploids followed by tetrad dissection indicate that it is essential for viability (Figure S3).

### Scm3 Is Required for Incorporation of CENP-A^Cnp1^ into Central Core Chromatin

The phenotypes of *scm3* mutants are suggestive of defective CENP-A^Cnp1^ chromatin integrity as previously seen in *cnp1*, *sim3*, and *sim4* mutants (Castillo et al., 2007; Dunleavy et al., 2007; Pidoux et al., 2003). We therefore examined whether CENP-A^Cnp1^ incorporation at centromeres is affected in *scm3* mutants. Following growth at 36°C, wild-type, *scm3-15*, *scm3-106*, and *scm3-139* cells were stained with anti-CENP-A^Cnp1^ and anti-Sad1 (a spindle pole body [SPB] protein [Hagan and Yanagida, 1995]). In G2, centromeres gather at the SPB on the nuclear periphery (Funabiki et al., 1993), forming a characteristic single spot of CENP-A^Cnp1^. In wild-type cells, CENP-A^Cnp1^ is detected as a single dot but is undetectable when Scm3^Sp^ function is compromised in *scm3* mutants (Figure 1D).

Consistent with this, chromatin immunoprecipitation (ChIP) shows that the level of CENP-A^Cnp1^ in subkinetochore chromatin (*cnt1*) is dramatically reduced in *scm3-15*, *scm3-106*, and *scm3-139* cells relative to wild-type cells at 36°C (Figure 1E). In wild-type cells the central domain is underrepresented in anti-histone H3 ChIP, and the level of H3 is increased in cells with reduced CENP-A^Cnp1^ at centromeres (Castillo et al., 2007). In keeping with this, a reciprocal increase in the levels of H3 associated with the central core region is observed in *scm3-106* (Figure 1F).

### Scm3^Sp^ Associates with Centromeres in a Cell-Cycle-Dependent Manner

The deposition of CENP-A could be influenced by many factors, including changes in the relative levels of histones, histone posttranslation modifications, nuclear import, defects in histone chaperones, or chromatin assembly factors. However, a protein that is required for CENP-A deposition, resides specifically at centromeres, and physically interacts with CENP-A is likely to play a direct and specific role in the assembly of CENP-A nucleosomes.

**Figure 2:**
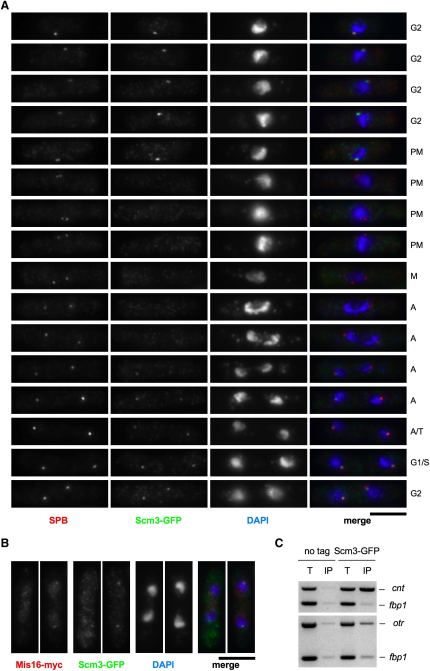
Scm3^Sp^ Is a Kinetochore Protein that Is Recruited in Late Anaphase (A) Immunofluorescence of cells expressing Scm3-GFP stained with antibodies to GFP (green) and Cdc11 (SPB; red) and DAPI (blue). Based on morphology and SPB separation, cells were assigned to cell cycle stages: G2; PM, premetaphase (prophase and prometaphase); M, metaphase; A, anaphase; T, telophase; G1; S phase. Scale bar, 5 μm. (B) Immunofluorescence of cells expressing Scm3-GFP and Mis16-myc stained with antibodies to GFP (green) and myc (red) and DAPI. Cells shown are in mid to late anaphase. (C) ChIP of Scm3-GFP using anti-GFP antibodies. Multiplex PCR indicates that Scm3-GFP is associated with central core domain (*cnt*) but not a euchromatic control locus (*fbp1*) or centromeric outer repeats (*otr*).

To examine the localization of Scm3^Sp^, the endogenous gene was fused with GFP. Our initial analyses indicated that Scm3^Sp^-GFP colocalizes with CENP-A^Cnp1^ during interphase, but in contrast to CENP-A^Cnp1^ it appears to dissociate from centromeres at the onset of mitosis and to reassociate in telophase (Figure S4). Single kinetochore signals are clearly seen for CENP-A^Cnp1^ during mitosis, whereas no signal is observed for Scm3^Sp^. Similar localization patterns were seen upon costaining with anti-Scm3^Sp^ antibodies (Figure S4). To pinpoint more precisely the timing of Scm3^Sp^ departure/arrival at centromeres, wild-type cells expressing Scm3^Sp^-GFP were stained with anti-Cdc11 antibodies to decorate SPBs (Krapp et al., 2001) and anti-GFP to detect Scm3^Sp^. Pole-to-pole separation allows the stages of mitosis to be determined; Scm3-GFP is at centromeres in G2 but dissociates just after spindle formation (SPB separation) and reassociates following sister-chromatid segregation to the poles, in mid-late anaphase B (Figure 2A). This temporal pattern of localization is reminiscent of that described previously for Mis16 and Mis18 (Fujita et al., 2007; Hayashi et al., 2004). Examination of cells expressing Scm3^Sp^-GFP and Mis16-myc revealed that they colocalize at centromeres and that their relative timing of reassociation in mitosis is similar (Figure 2B). This and previous analyses (Fujita et al., 2007) suggest that dissociation of Scm3^Sp^, Mis16, and Mis18 from centromeres is coincident with spindle formation in early mitosis and that they reassemble at centromeres in mid-anaphase B after chromosome disjunction. Thus, Scm3^Sp^, Mis16, and Mis18 may function together. This dynamic behavior contrasts with that of CENP-A^Cnp1^, which remains at centromeres throughout mitosis (Figure S4) (Takahashi et al., 2000; Takayama et al., 2008).

The association of Scm3^Sp^-GFP with centromeres was also analyzed by ChIP with anti-GFP antibodies. Like CENP-A^Cnp1^, Scm3^Sp^-GFP is enriched in the central domain (*cnt1*; Figure 2C) but not in the centromeric outer repeats. Using primer pairs spaced across the entire ∼12 kb central domain of *cen1*, it is clear that Scm3^Sp^-GFP is not restricted to discrete pockets within the central domain, but like CENP-A^Cnp1^ is distributed across the entire domain (Figure S5).

**Figure 3:**
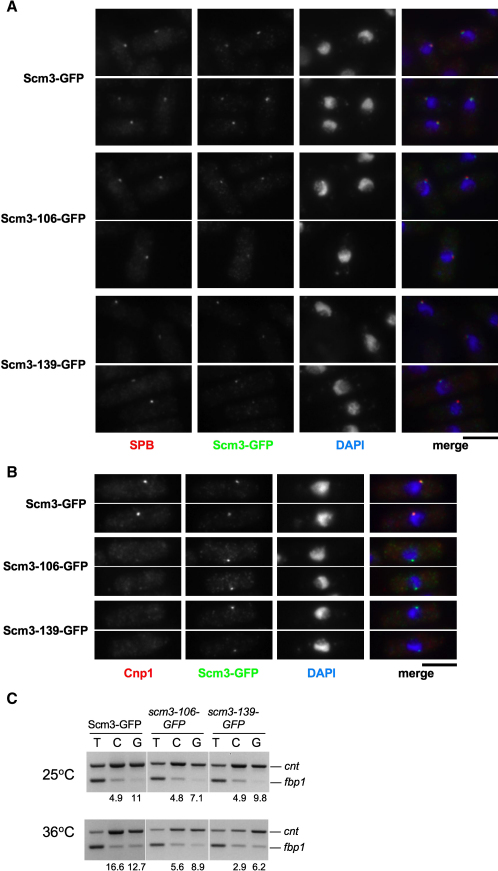
Scm3^Sp^ Mutant Proteins Remain Localized at the Centromere, but CENP-A^Cnp1^ Is Lost (A) Strains expressing Scm3-GFP, Scm3-106-GFP, or Scm3-139-GFP were shifted to 36°C for 6 hr, fixed, and processed for immunofluorescence using anti-GFP (Scm3-GFP; green), anti-Cdc11 antibodies (SPB; red), and DAPI (blue). Identical exposures and processing were performed, to ensure that wild-type and mutant images are comparable. All cells are in G2. Scale bar, 5 μm. (B) As (A), except antibodies are anti-GFP (green) and anti-Cnp1 (red). (C) Strains expressing Scm3-GFP, Scm3-106-GFP, and Scm3-139-GFP were shifted to 36°C for 6 hr, fixed, and analyzed by ChIP with anti-Cnp1/CENP-A^Cnp1^ (C) and anti-GFP (G, Scm3-GFP) antibodies. PCR analysis as in Figure 1E.

The *scm3-106* and *scm3-139* alleles were also fused to GFP. Examination of fixed cells indicated that Scm3^Sp^-106-GFP and Scm3^Sp^-139-GFP mutant proteins remain localized at centromeres (Figure 3A), though in some cells a reduction in signal intensity was observed. Mutant Scm3 is recruited to centromeres, even though CENP-A^Cnp1^ localization is clearly lost (Figure 3B). ChIP confirms that Scm3^Sp^-106-GFP and Scm3^Sp^-139-GFP mainly remains at centromeres even when CENP-A^Cnp1^ levels are reduced (Figure 3C). Thus, mutant Scm3 protein appears to be defective in mediating the deposition of CENP-A^Cnp1^, despite the fact that it localizes at centromeres.

Importantly, this observation, and the fact that Scm3^Sp^-GFP dissociates from centromeres in mitosis while CENP-A^Cnp1^ remains, uncouple Scm3^Sp^ and CENP-A^cnp1^ localization from each other and indicate that Scm3^Sp^ may not be an integral component of a CENP-A-H4-Scm3^Sp^ variant nucleosome in fission yeast.

### CENP-A^Cnp1^ Extraction from Chromatin Is Distinct from Scm3^Sp^

Analyses in budding yeast have suggested that Scm3^Sc^ forms part of an unusual single nucleosome that contains CENP-A^Cse4^, H4, and Scm3^Sc^, but no H2A-H2B. It has been proposed that this may represent a key feature of subkinetochore nucleosomes (Mizuguchi et al., 2007). As described above, Scm3^Sp^ association with and release from centromeres is not directly linked to the formation of CENP-A^Cnp1^nucleosomes.

We performed ChIP on cells expressing H2B-FLAG (Zofall and Grewal, 2007) and confirmed that it is detectable in the central core as shown previously (Maruyama et al., 2006). However, by using primers across centromere 1 we observe that H2B-FLAG enrichment follows a similar pattern to H3 (Figure S7), which has been shown to be underrepresented in the central domain compared to euchromatin (Castillo et al., 2007). This observation suggests that CENP-A^Cnp1^ nucleosomes may lack H2A/B, although there may be other explanations (see Discussion).

**Figure 4:**
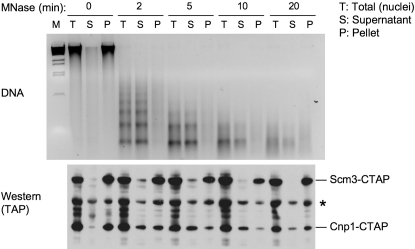
Scm3^Sp^ Does Not Extract with CENP-A^Cnp1^ from Chromatin Extraction of CENP-A^Cnp1^ from chromatin by MNase digestion. Nuclei were prepared from cells in which endogenous *scm3*^+^ and *cnp1*^+^ genes were TAP-tagged. After progressive MNase digestion as labeled, total nuclei digestion mixtures (T) were separated into soluble supernatant (S) and nuclei pellets (P) by centrifugation. Samples were subject to DNA extraction and electrophoresis (upper panel) or protein extraction and western blotting (lower panel). Bands labeled with an asterisk are nonspecific proteins detected by the antibody. Figure S8 shows that bulk histones (H4) are also released into supernatant.

To investigate further whether unusual nucleosomes such as those proposed for *S. cerevisiae* might exist at fission yeast centromeres, we performed a MNase digestion time course on nuclei and, following centrifugation, assessed the relative proportions of Scm3^Sp^-TAP and CENP-A^Cnp1^-TAP released from the insoluble chromatin pellet into the soluble fraction. Most CENP-A^Cnp1^-TAP and bulk histones are released into the pellet after 2–10 min of MNase treatment, whereas Scm3^Sp^-TAP remains tightly associated with the pellet even after 20 min digestion (Figures 4 and S8). This demonstrates that Scm3^Sp^ and CENP-A^Cnp1^ do not extract from chromatin under the same conditions; Scm3^Sp^-TAP remains insoluble, whereas CENP-A^Cnp1^ is released into the supernatant along with oligomeric nucleosome particles and other histones. Taken together, our analyses suggest that Scm3^Sp^ might act to assemble CENP-A^Cnp1^ into subkinetochore chromatin at these regional centromeres, but it is unlikely to be an integral component of CENP-A^Cnp1^ nucleosomes themselves in fission yeast.

### Scm3 Localization Requires Sim4, Mis6, Mis16, and Mis18, but Not CENP-A^Cnp1^

**Figure 5:**
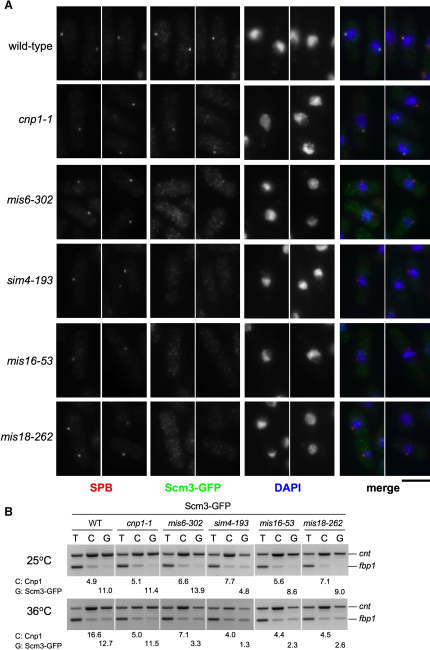
Localization of Scm3^Sp^ Is Dependent on Mis6, Sim4, Mis16, Mis18, but Not CENP-A^Cnp1^ (A) Wild-type, *cnp1-1*, *mis6-302*, *sim4-193*, *mis16-53*, and *mis18-262* strains expressing Scm3-GFP were shifted to 36°C for 6 hr, fixed, and processed for immunofluorescence using anti-GFP (Scm3-GFP; green), anti-Cdc11 antibodies (SPB; red), and DAPI (blue). Identical exposures and processing were performed to ensure that wild-type and mutant images are comparable. Representative images are presented. All cells are in G2. Scale bar 5 μm. (B) ChIP of Scm3-GFP in the indicated strains shifted to 36°C for 6 hr before fixation and analysis by ChIP with anti-Cnp1/CENP-A^Cnp1^ (C) and anti-GFP (G, Scm3-GFP) antibodies. PCR analysis as in Figure 1E. Part of same experiment shown in Figure 3C; wild-type control identical.

**Figure 6:**
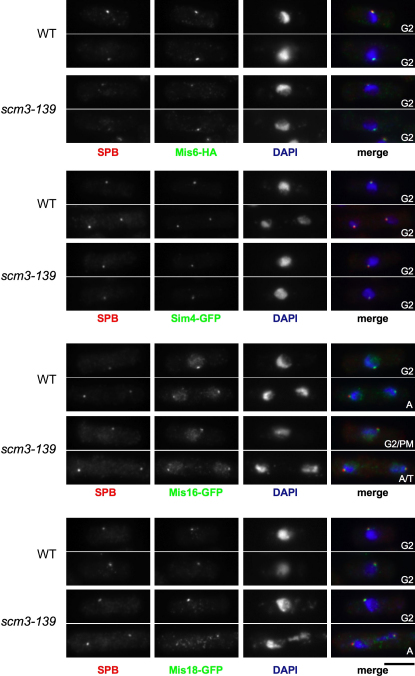
Kinetochore Proteins Remain Localized in *scm3* Mutants Wild-type and *scm3-139* mutants expressing Mis6-HA, Sim4-GFP, Mis16-GFP, and Mis18-GFP were shifted to 36°C for 6 hr, fixed, and processed for immunofluorescence. For Mis6-HA cells antibodies are SPB (anti-Sad1; red) and anti-HA (Mis6-HA: green). For GFP-tagged strains antibodies are SPB (anti-Cdc11; red) and anti-GFP (Sim4, Mis16, Mis18: green). DAPI is in blue. Identical exposures and processing were performed to ensure that wild-type and mutant images are comparable. Representative images are presented. Cells were assigned to cell-cycle stages (see Figure 2A legend). Scale bar, 5 μm.

As Scm3^Sp^, Mis16, and Mis18 display highly similar temporal localization during the cell cycle, we investigated dependency relationships between these proteins. We examined whether Scm3^Sp^ localization is affected by temperature-sensitive conditional mutations in Mis16 and Mis18 and other kinetochore proteins including CENP-A^Cnp1^, Sim4, and Mis6 (Figure 5A). Reciprocally, we determined if the localization of Mis6-HA, Sim4-GFP, Mis16-GFP, or Mis18-GFP is disrupted in the *scm3-139* mutant (Figure 6). Scm3^Sp^-GFP is lost from centromeres in *mis16-53* and *mis18-262* mutants and also in *sim4-193* and *mis6-302* at 36°C. However, in cells with defective CENP-A^Cnp1^ (*cnp1-1*), which have dramatically reduced levels of CENP-A^Cnp1^ at centromeres, Scm3^Sp^-GFP remains at centromeres. Cells with defective Mis12, or that lack CENP-C^Cnp3^ (*cnp3*Δ), or the CENP-A^Cnp1^ escort Sim3 (*sim3*Δ), also retain Scm3^Sp^-GFP at centromeres (Figure S9). This indicates that the maintenance of Scm3^Sp^ at centromeres is independent of CENP-A^Cnp1^-containing chromatin but that its localization is dependent on other kinetochore proteins (Sim4 and Mis6) known to affect the maintenance of CENP-A^Cnp1^ at centromeres. This is underscored by the fact that individual cells with strong Scm3^Sp^-GFP staining but undetectable CENP-A^Cnp1^ are common in *cnp1-1* (Figure S6 and data not shown). In agreement with these localization data, ChIP analyses shows that Scm3^Sp^-GFP levels drop significantly in the central domain in *sim4-193*, *mis6-302*, *mis16-53*, and *mis18-262*, but not in *cnp1-1* (Figure 5B).

Mis6-HA, Sim4-GFP, Mis16-GFP, and Mis18-GFP were found to remain at centromeres in *scm3-139* cells at 36°C (Figure 6). Thus, the localization to centromeres of the three proteins (Mis16, Mis18, and Scm3^Sp^) that display dynamic behavior in mitosis is unaffected by defects in Scm3^Sp^. These analyses suggest that Mis18 and Mis16 are required to recruit Scm3^Sp^ to centromeres but that loss of Scm3^Sp^ function does not affect the recruitment of Mis16, Mis18, or Scm3^Sp^. However, these three proteins are required to maintain CENP-A^Cnp1^ at centromeres (Figures 1D and 1E above; Hayashi et al., 2004), suggesting that the recruitment of Scm3^Sp^ by Mis16-Mis18 to the central domain is critical for the incorporation of CENP-A^Cnp1^ in place of H3.

In addition, we have observed synthetic phenotypes (synthetic lethality or growth retardation) between *scm3 (sim1)* mutants and *cnp1*, *sim3*, *sim4*, *mis6*, *mis16*, and *mis18* mutants (Pidoux et al., 2003; and data not shown). Overexpression of CENP-A^Cnp1^ suppresses *scm3*, *mis16*, *mis18*, *sim4*, *mis6*, and *sim3* mutants (Pidoux et al., 2003; Hayashi et al., 2004), and overexpression of Scm3^Sp^ suppresses *sim3* and *sim4* mutants (Pidoux et al., 2003). These genetic interactions are strongly suggestive of functional interactions between Scm3^Sp^ and other proteins involved in CENP-A^Cnp1^ assembly.

### Scm3^Sp^ Physically Associates with CENP-A^Cnp1^ and Mis18

**Figure 7:**
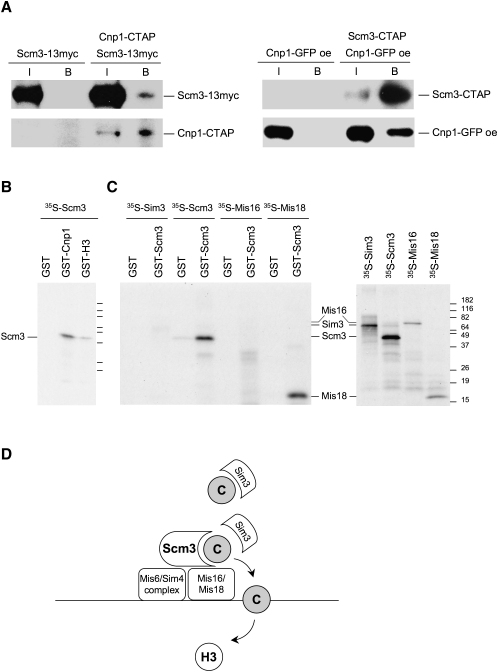
Scm3^Sp^ Associates with Itself, CENP-A^Cnp1^, and Mis18 (A) Coaffinity purification of Scm3^Sp^ and CENP-A^Cnp1^. Strains expressing the following tagged protein(s) were analyzed: Scm3-13myc only, both Scm3-13myc and CENP-A^Cnp1^-CTAP, CENP-A^Cnp1^-GFP only, or both CENP-A^Cnp1^-GFP and Scm3-CTAP. Tagged genes were expressed with the native promoters at their endogenous loci, except that CENP-A^Cnp1^-GFP was overexpressed (labeled with “oe”). IgG beads were used for affinity purification from cell extracts. Input samples (I) and bead-bound samples (B) were subject to western blotting using the indicated antibodies. (B) In vitro binding assay. (Left) ^35^S-labeled Scm3^Sp^ was produced by in vitro transcription-translation in reticulocyte lysate (see [C], right panel) and incubated with GST, GST-Cnp1, or GST-H3. Complexes were pulled down with glutathione agarose, washed, and analyzed by SDS-PAGE and fluorography. (C) ^35^S-labeled Sim3, Scm3^Sp^, Mis16, and Mis18 were produced (right panel) and used in vitro binding assay with GST or GST- Scm3^Sp^ (left panel). (D) Model for Scm3^Sp^ function. The Mis6/Sim4 complex and Mis16/Mis18 are required to recruit Scm3^Sp^ to centromeres. Scm3^Sp^ acts as a receptor at the centromere for incoming CENP-A^Cnp1^ from the Sim3 escort-chaperone. In conjunction with Mis16/Mis18 and other factors, Scm3^Sp^ mediates the incorporation of CENP-A^Cnp1^ (C) in subkinetochore chromatin in place of H3.

Mis16 and Mis18 have been reported to coimmunoprecipitate, indicating that they associate in a complex (Hayashi et al., 2004). However, although Mis16 and Mis18 affect CENP-A^Cnp1^ recruitment to centromeres, neither has been found in association with CENP-A^Cnp1^. A possible role for Scm3^Sp^ might be to act as the receptor for CENP-A^Cnp1^ at the central domain of centromeres after delivery by the Sim3 escort (Dunleavy et al., 2007). This scenario predicts that Scm3^Sp^ would interact with CENP-A^Cnp1^. In strains expressing CENP-A^Cnp1^-TAP and Scm3^Sp^-myc, from their own promoter at the endogenous locus, Scm3^Sp^-myc is pulled down with CENP-A^Cnp1^-TAP. Reciprocally, in Scm3^Sp^-TAP strains overexpressing GFP-tagged CENP-A^Cnp1^, CENP-A^Cnp1^-GFP is pulled down with Scm3^Sp^-TAP (Figure 7A). Scm3^Sp^-myc is also pulled down with sheep anti-Cnp1 serum (Figure S10). Interestingly, in cells lacking the CENP-A^Cnp1^ escort Sim3 (*sim3*Δ), the interaction between Scm3-TAP and overexpressed CENP-A^Cnp1^-GFP is disrupted, suggesting that Sim3 is required to facilitate interactions between CENP-A^Cnp1^ and Scm3^Sp^ (Figure S10). This may be consistent with the Sim3 handing over CENP-A^Cnp1^ to Scm3^Sp^.

To determine if Scm3^Sp^ can directly interact with CENP-A^Cnp1^, we tested if ^35^S-labeled Scm3^Sp^ can associate in vitro with recombinant GST, GST-H3, and GST-CENP-A^Cnp1^. ^35^S-Scm3^Sp^ is pulled down with GST-CENP-A^Cnp1^ more efficiently than with GST-H3 (Figure 7B). These analyses suggest that Scm3^Sp^ may have a preference for binding CENP-A^Cnp1^ but can bind H3. Interactions with other histones are not unexpected if Scm3^Sp^ is involved in events that remove H3 and deposit CENP-A^Cnp1^ in its place.

To determine if Scm3^Sp^ can physically interact with itself, we incubated ^35^S-Scm3^Sp^ with GST-Scm3^Sp^; Scm3^Sp^ self-associates (Figure 7C), consistent with the dimerization reported for *S. cerevisiae* Scm3^Sp^ (Mizuguchi et al., 2007). In addition, we find that ^35^S-Mis18, but not ^35^S-Mis16 or ^35^S-Sim3, can associate with GST-Scm3^Sp^ in vitro (Figure 7C). This may indicate that Scm3^Sp^ can be recruited to centromeres by direct association with Mis18.

## Discussion

The point centromeres of *S. cerevisiae*, formed around a single CENP-A^Cse4^ nucleosome, are the most thoroughly dissected and understood (Westermann et al., 2007). Our comprehension of regional centromeres containing arrays of CENP-A nucleosomes, such as those of fission yeast and metazoa, is less well developed. However, genetic and affinity purification approaches have had a major impact on defining their protein composition (Foltz et al., 2006; Hayashi et al., 2004; Liu et al., 2005; Obuse et al., 2004a, 2004b; Okada et al., 2006; Pidoux et al., 2003). *S. cerevisiae* Scm3^Sc^ was originally identified as a multicopy suppressor of a histone fold mutation in CENP-A^Cse4^ (Chen et al., 2000; Stoler et al., 2007). Scm3^Sc^ was shown to affinity purify with CENP-A^Cse4^ and to be a component of kinetochore chromatin (Camahort et al., 2007; Mizuguchi et al., 2007). We identified fission yeast Scm3^Sp^ through a screen for mutants defective in marker gene silencing in the central domain of a fission yeast centromere (Pidoux et al., 2003). Our analyses demonstrate that Scm3^Sp^ plays a critical and conserved role at regional centromeres where it is required to maintain CENP-A^Cnp1^ chromatin and kinetochore integrity to allow normal chromosome segregation.

In common with other proteins identified in the central domain silencing screen such as Sim4, Sim3, and CENP-A^Cnp1^ itself, Scm3^Sp^ (Sim1) function is required for normal amounts of CENP-A^Cnp1^ at centromeres. In restrictive conditions, *scm3* mutants have dramatically reduced CENP-A^Cnp1^ levels at centromeres, and a concomitant increase in H3 (Figure 1). This phenotype is consistent with a role for Scm3^Sp^ in either assembly or maintenance of CENP-A^Cnp1^ at centromeres. In support of a role in CENP-A^Cnp1^ assembly, analyses indicate that Scm3 is required for incorporation of newly synthesized CENP-A^Cnp1^ (Figure S11), although this could also be interpreted as ineffective retention of new CENP-A^Cnp1^ once assembled. Sim3 (a NASP/N1-N2 ortholog) is a soluble nucleoplasmic protein that associates with CENP-A^Cnp1^ and may escort CENP-A^Cnp1^ to centromeres (Dunleavy et al., 2007). In contrast, we have shown that Scm3^Sp^ is a centromere component residing in the subkinetochore central domain. Unlike the majority of centromere proteins in fission yeast that remain centromere associated throughout the cell cycle, Scm3^Sp^ dissociates from the centromere upon entry into mitosis and returns in late anaphase. Consistent with this, the accompanying paper demonstrates that Scm3 is absent from central core chromatin in mitotically blocked cells (Williams et al., 2009 [this issue of *Molecular Cell*]). Thus, Scm3^Sp^ displays the same cell-cycle-dependent pattern of localization as Mis16 and Mis18. Human Mis18 and Mis18BP/KNL2 proteins also associate with centromeres in telophase/early G1 and precede CENP-A^Cnp1^ deposition upon mitotic exit (Fujita et al., 2007; Jansen et al., 2007; Maddox et al., 2007). Each of these proteins has been shown to be required for CENP-A localization at centromeres and have been placed at the top of the hierarchy of kinetochore proteins. However, none of these proteins have been found to associate with CENP-A.

We have shown that Mis16 and Mis18 are required for Scm3^Sp^ association with centromeres. Conversely, Scm3^Sp^ is not required for localization of Mis16 and Mis18. In addition, Scm3^Sp^ can associate with both CENP-A^Cnp1^ and Mis18, and the accompanying paper reports a 2-hybrid interaction between Scm3^Sp^ and Mis16 (Williams et al., 2009). Thus, genetic and biochemical evidence suggests that Scm3^Sp^ could be the link between Mis16/Mis18 and CENP-A^Cnp1^ and its deposition.

Proteins of the Mis6 complex are dependent on Mis16 and Mis18 for centromere association (Hayashi et al., 2004), even though Mis6 complex proteins are located at the centromere throughout the cell cycle. One interpretation is that Mis16 and Mis18 are required for their recruitment but not retention at the centromere, and thus Mis16 and Mis18 are released in mitosis without affecting Mis6, Sim4, or other components. Both *mis6* and *sim4* mutants display a dramatic decrease in CENP-A^Cnp1^ levels at centromeres. Proteins of the orthologous CENP-H/I complex in vertebrate cells have also been shown to affect localization of CENP-A (Okada et al., 2006). We find that Scm3^Sp^ localization is dependent on both Mis6 and Sim4. Our observations suggest that Scm3^Sp^ (in conjunction with other proteins such as Mis16 and Mis18) is the mediator of CENP-A^Cnp1^ deposition and the protein through which the CENP-A^Cnp1^-depletion phenotypes of *mis16*, *mis18*, *sim4*, and *mis6* mutants are expressed. The defect in Scm3 mutants is not due to an inability to localize—they remain at centromeres while CENP-A^Cnp1^ is delocalized—suggesting that they are defective in the interaction with, or deposition of, CENP-A^Cnp1^. Among Scm3^Sp^, Mis16, and Mis18, only Scm3^Sp^ has been found to bind CENP-A^Cnp1^; it is a good candidate for being a centromere-associated receptor or assembly factor for incoming CENP-A. Our data point to a model in which Mis16 and Mis18 are required to recruit Scm3^Sp^, which acts as a receptor for CENP-A^Cnp1^ and ensures its deposition at centromeres (Figure 7D).

The fact that histone H3 levels increase at centromeres in cells with impaired Scm3^Sp^ function suggests that it is required for the replacement of histone H3 with CENP-A^Cnp1^. This may occur coincident with centromere replication or in G2 (Castillo et al., 2007; Takayama et al., 2008). At *S. cerevisiae* centromeres, H2A and H2B have been found to be diminished, and in vitro Scm3^Sc^ can cause the release of H2A and H2B from preformed CENP-A^Cse4^ octamers to form a stable 1:1:1 hexameric complex with CENP-A and H4. Thus, it has been proposed that Scm3^Sc^ participates in the formation of a unique type of CENP-A^Cse4^ nucleosome at *S. cerevisiae* centromeres and that similar irregular CENP-A nucleosomes might play a pivotal role at regional centromeres (Mizuguchi et al., 2007). Our and other analyses indicate that CENP-A^Cnp1^ is enriched within the central domain (∼12 kb) at fission yeast centromeres (Takahashi et al., 2000). Reciprocally, H3 is underrepresented (Castillo et al., 2007). Our ChIP analysis suggests that H2B is similarly reduced (Figure S7), and in agreement, the accompanying paper shows that H2A and H2B as well as H3 are highly diminished in the central core domain, and that this is dependent on Scm3^Sp^ function (Williams et al., 2009). These observations are consistent with the interpretation that CENP-A^Cnp1^ nucleosomes lack H2A-H2B, although it is also possible that the lower apparent enrichment might instead reflect a less stable association of H2A-H2B dimers with CENP-A^Cnp1^ nucleosomes compared to canonical H3 nucleosomes (Henikoff, 2008). From what is known about the process of nucleosome disassembly and reassembly, it is likely that the replacement of H3 with CENP-A may initiate with the release of H2A-H2B to allow access and removal of H3 (Williams and Tyler, 2007). At *S. cerevisiae* centromeres, H3 was found to increase at centromeres in the absence of Scm3^Sc^ (Camahort et al., 2007; although this was not detected by others, Mizuguchi et al., 2007), suggesting that Scm3^Sc^ may aid replacement of H3 with CENP-A^Cse4^. It is possible that the mechanism by which Scm3^Sc^, in cooperation with Ndc10, promotes CENP-A^Cse4^ assembly at *S. cerevisiae* centromeres operates to effectively trap a CENP-A^Cse4^-H4 tetrameric intermediate in the process of remodeling an H3-containing octamer and it is this that remains associated with Scm3^Sc^ and tethered to centromere DNA via Ndc10. In other organisms such as fission yeast with regional centromeres, the process may remain dynamic, allowing some H2A-H2B reassociation following deposition of CENP-A in place of H3. In *Drosophila* and human cells, most CENP-A nucleosomes have been reported to contain CENP-A, H4, H2A, and H2B, which may be octamers or tetrameric hemisomes (Blower et al., 2002; Dalal et al., 2007; Foltz et al., 2006).

Scm3-like proteins can be readily identified in fungi, but it has not been possible to recognize orthologs in more complex eukaryotes (Aravind et al., 2007). Since centromeric DNA and the CENP-A variant itself are rapidly evolving, it is conceivable that Scm3-related proteins exist in metazoans but are not currently recognizable. The identification of proteins occupying the same functional niche may eventually resolve this. Our analyses indicate that Scm3^Sp^ is unlikely to be part of an unusual CENP-A^Cnp1^ nucleosome in fission yeast, as CENP-A^Cnp1^ behavior can be uncoupled from that of Scm3^Sp^: first, Scm3^Sp^ is released at mitosis, whereas CENP-A^Cnp1^ remains centromere associated; second, Scm3^Sp^ remains chromatin associated under conditions that release nucleosome particles and solubilize most CENP-A^Cnp1^; third, CENP-A^Cnp1^ is lost from centromeres in *scm3* mutants but the mutant Scm3^Sp^ protein remains centromere associated; and fourth, in the CENP-A^Cnp1^ mutant *cnp1-1*, where centromeres are rendered nonfunctional, most CENP-A^Cnp1^ is lost but Scm3^Sp^ remains at centromeres.

Fission yeast Scm3^Sp^ clearly behaves differently from *S. cerevisiae* Scm3 in that its association with centromeres is cell cycle dependent. It seems reasonable to propose that a Mis16-Mis18-Scm3^Sp^ complex is formed at fission yeast centromeres and this facilitates the deposition of CENP-A^Cnp1^. Fission yeast CENP-A^Cnp1^ has been shown to be deposited during S and G2 stages of the cell cycle (Dunleavy et al., 2007; Takayama et al., 2008), raising the issue of why the Mis16, Mis18, and Scm3 proteins are released from centromeres between early mitosis and anaphase B. Several putative CDK phosphorylation sites are present in the Mis16, Mis18, and Scm3^Sp^ proteins. It is possible that, when CDK levels are high upon entry into mitosis, Mis16, Mis18, or Scm3^Sp^ become phosphorylated and this simply leads to their dissociation; dephosphorylation following anaphase would allow them to rebind. Indeed, we have found that at least serine 127 of Scm3^Sp^ is phosphorylated at a putative CDK site, although it is not known if this modification is cell cycle regulated or that it regulates Scm3^Sp^ localization (Figures 1C and S12). The dissociation/reassociation of Mis16, Mis18, and Scm3^Sp^ might represent a key regulatory event in the cell cycle; the successful segregation of sister centromeres to opposite poles in anaphase might be a signal that subsequently permits the rebinding of Mis16, Mis18, and Scm3^Sp^. This could act to link CENP-A replenishment at a particular centromere to the performance of that kinetochore in mitosis (Ahmad and Henikoff, 2002; Carroll and Straight, 2006; Mellone and Allshire, 2003). However, experiments in human cells indicate that CENP-A is replenished in cells that complete an aberrant mitosis without microtubules in the absence of the spindle assembly checkpoint (Jansen et al., 2007). Thus, there is currently no evidence that a marking system operates during mitosis to permit CENP-A deposition at centromeres that have functioned correctly in terms of achieving a bioriented state and segregating accurately.

It is conceivable that the cell-cycle-regulated localization of Mis16, Mis18, and Scm3^Sp^ may just be a passive reflection of other events that alter kinetochore configuration upon chromosome condensation in mitosis to accommodate other proteins involved in making and monitoring microtubule attachments. However, the regulation of Mis18 and associated proteins during the cell cycle is conserved. In human cells, the orthologs Mis18 (Mis18α and Mis18β) and the associated Mis18BP/KNL2 proteins only associate with centromeres for a brief period between telophase and mid-G1 (Fujita et al., 2007; Maddox et al., 2007). Interestingly, the deposition of CENP-A is normally only observed in this same window of the cell cycle, immediately after mitotic exit in early G1 (Jansen et al., 2007). Since hMis18α, hMis18β, and Mis18BP are critical for CENP-A deposition, this suggests that the recruitment of these Mis18 proteins is required for the incorporation of CENP-A into subkinetochore chromatin.

In fission yeast, Mis16, Mis18, and Scm3^Sp^ associate with centromeres from anaphase B through S phase and G2 but are released in early mitosis. CENP-A^Cnp1^ is incorporated at fission yeast centromeres in S phase and G2 phases of the cell cycle (Takayama et al., 2008). Although it appears that the timing of CENP-A deposition differs between fission yeast and metazoa, in a normal fission yeast cell cycle, G1 is extremely short, with G1 and S phases occurring rapidly upon exit from mitosis (at the time of cytokinesis), meaning that CENP-A^Cnp1^ deposition also closely follows mitosis in fission yeast. The return of Scm3 to centromeres in late mitosis may be necessary and permissive for CENP-A^Cnp1^ assembly, but association of another factor or a modification later in the cell cycle may additionally be required for it to actually happen. Regardless of the specifics of timing in different systems, recruitment of Mis18 and associated proteins such as Scm3^Sp^ or hMis18BP appears to be a key conserved step in mediating CENP-A deposition.

## Experimental Procedures

### Cell Growth and Manipulation

Standard genetic and molecular techniques were followed. Fission yeast methods were as described (Moreno et al., 1991).

### Identification of the *sim1^+^/scm3^+^* Gene

For cloning of *sim1*^+^/*scm3^+^* ORF, *sim1-106* was transformed with a genomic library (pDB), and complementing plasmids were identified and partially sequenced. At the time, the *S. pombe* genome sequence was incomplete, and further sequencing of the region was performed by the Sanger Centre. This allowed identification of SPAPB1A10.02 as the *sim1*^+^ ORF. Mutant alleles were sequenced by PCR amplification from mutant genomic DNA. Although 11 *sim1* mutant alleles were identified in the original *sim* screen, sequencing revealed that these comprised only three different mutations. As each of the 11 mutants was independently isolated, this suggests either that the mutants were pre-existing in the population before mutagenesis (by UV of *S. pombe* on plates), or that these mutations particularly strongly induce the desired phenotype.

For tagging of endogenous *scm3* with GFP, an ∼500 bp fragment of the 3′ end of the ORF was amplified by PCR and cloned into pDM84 (see Dunleavy et al., 2007). The resultant plasmid was linearized with BglII and transformed into *S. pombe*. Correct integration was confirmed by PCR and sequencing. As the mutations in *scm1-106* and *scm3-139* are near the N terminus, this method was also used to tag these mutant alleles.

### ChIP

If appropriate, cells were shifted to restrictive temperature (36°C) for 6 hr before fixation. The H2B-FLAG was a gift from Shiv Grewal (Zofall and Grewal, 2007). ChIP was performed as described (Pidoux et al., 2003; Castillo et al., 2007; Dunleavy et al., 2007), or with the following modifications. Cells were fixed with 1% PFA for 20 min at room temperature and lysed with Fastprep (3 times 20 s at maximum speed). Extracts were centrifuged for 15 min at 14,000 rpm at 4°C to purify insoluble fraction (pellet), which is enriched with chromatin. The supernatant containing the soluble fraction was discarded and the pellet was washed twice with lysis buffer. Chromatin was solubilized by shearing with the Bioruptor sonicator (24 min, 30 s On and 30 s Off at “High” [200 W] position). For immunoprecipitation, 10 μl of anti-Cnp1 antiserum, 3 μl of anti-H3C antibody (Abcam, ab1791), or 1.5 μl of anti-GFP antibody (Invitrogen, A-11122) and either protein G (for anti-CENP-A^Cnp1^) or protein A (for anti-H3C and anti-GFP) Sepharose beads were used. ChIPs were analyzed by multiplex PCR and quantification of ethidium bromide-stained gels, as described (Pidoux et al., 2003; Castillo et al., 2007).

### Cytology

Immunolocalization was performed as described (Pidoux et al., 2003; Castillo et al., 2007). If appropriate, cells were shifted to restrictive temperature (36°C) for 6 hr before fixation. Cells were fixed for 7–10 min with 3.7% formaldehyde. Fixation of cells for tubulin staining used formaldehyde and 0.05% glutaraldehyde as described (Dunleavy et al., 2007). Antibodies used were kind gifts of I. Hagan (Sad1; 1:10), K. Gull (TAT1 anti-tubulin; 1:15), I. Samejima/K. Sawin (anti-Cdc11; 1:3000), K. Samejima (12CA5 anti-HA; 1:300), and K. Hardwick (anti-GFP; 1:500). Anti-CENP-A^Cnp1^ antiserum was used at 1:1500. 9E10 anti-myc (Covance) was used at 1:5000. Alexa Fluor 594- and 488-coupled secondary antibodies were used at 1:1000 (Invitrogen). Microscopy was performed using a Zeiss Imaging 2 microscope using a 100× 1.3 NA Plan-Apochromat objective. Image acquisition was controlled using Metamorph software (Universal Imaging Corporation). Identical exposures were used for different strains in the same experiment. For display of images, maximum intensity was determined for, for example, Cnp1 staining in wild-type, and this maximum was applied for scaling of all wild-type and mutant images. FITC and TRITC channels were scaled in this way; DAPI images were autoscaled.

### In Vitro Binding Assays

In vitro binding experiments were performed as described (Dunleavy et al., 2007) except that pull-downs were performed in cold PBS containing 5 mg/ml BSA. *mis16*^+^ and *mis18*^+^ ORFs were PCR amplified from cDNA.

### Nucleosome Preparation

An established procedure was used for nucleosome preparation (Song et al., 2008). The nuclei were digested by 100 U/ml of micrococcal nuclease (Roche) for 5 min at 36°C. The reaction was stopped by adding modified TAP buffer (1 mM DTT, 5 mM EDTA, 5 mM EGTA, 50 mM NaCl, 10 mM Tris-HCl [pH 8.0], 0.1% NP40). The mono or dinucleosomes were confirmed by electrophoresis.

### Coimmunoprecipitation of Scm3 and CENP-A^Cnp1^

A modified of a previously described procedure was used to prepare cytoplasmic and nuclear extract (Bhargava et al., 1972). Immunoprecipitation from extracts were performed with Rabbit IgG-agarose (A2909, Sigma). After binding for 2∼4 hr at 4°C, the beads were washed three times with IPP150 buffer (10 mM Tris-Cl [pH 8.0], 150 mM NaCl, and 0.1% NP40) and eluted in SDS buffer (1% SDS, TE). Commercial antibodies used for western blotting included C-myc (9E10; Santa Cruz), GFP (B-2) (sc-9996; Santa Cruz), and Peroxidase-antiperoxidase (PAP) antibody (P-1291; Sigma). Western blots were developed using ECL reagents (Amersham Biosciences). For the pull-down of Sim1-13myc using sheep anti-Cnp1 serum (Figure S8), immunoprecipitation was performed as described (Dunleavy et al., 2007).

### Immunoaffinity Purification

Immunoaffinity purifications were performed essentially as described (Oeffinger et al., 2007) with the following modifications: *S. pombe* cultures were grown to the cell density of 10^7^ cells/ml in PMG complete media supplemented with 1 μM thiamine to partially repress the expression of Scm3-GFP fusion protein. For each sample, 7.5 g of cells, milled in solid phase, were used. Immunoprecipitations were performed using the protein A Dynabeads coupled to anti-GFP antibody (Invitrogen, A11122) for 90 min. After washes Dynabeads with immunoprecipitated material were subjected to on-bead Tryptic digestion. After the digestion, samples were acidified by adding TFA to a final concentration of 0.1% and spun onto StageTips as described elsewhere (Rappsilber et al., 2003, 2007). Peptides were eluted in 20 μl of 80% acetonitrile and 0.5% acetic acid and were concentrated to 2 μl (Concentrator 5301, Eppendorf AG). They were then diluted to 5 μl by 0.1% TFA and injected for LC-MS/MS analysis.

### Analysis of Incorporation of Newly Synthesized GFP-Cnp1

Induction was performed as described (Dunleavy et al., 2007), with modifications. Wild-type (FY8481) and scm3-139 (FY12454, 12455) strains were taken from YES plates containing 10% glucose and grown overnight at 25°C (permissive temperature) to mid-log-phase in PMG containing 10% glucose to maintain repression of the *inv1* promoter. Cells were then washed and transferred to normal PMG (2% glucose) and grown at 32°C (restrictive temperature for *scm3-139*). After 3 hr, cells were washed with dH_2_O and transferred to PMG containing 4% sucrose to induce the expression of GFP-Cnp1 from the *inv1* promoter. Wild-type cultures were observed periodically by live-cell fluorescence microscopy to determine when GFP-Cnp1 became expressed and localized at centromeres. When approximately 70% of wild-type cells showed a bright spot at centromeres, an aliquot of cells from wild-type and *scm3-139* cultures was fixed with 3% formaldehyde for 7 min (induced time point 1; total of 6 hr at 32°C). Additional time points were taken after an additional 30 and 60 min (induced time points 2 and 3). Cells maintained in repressed conditions (PMG 2% glucose) were also analyzed. Cells were examined by fluorescence microscopy (described elsewhere in manuscript). Images were acquired using identical exposures, scaling, and processes to ensure that they are directly comparable. Samples were also taken for western blotting to confirm expression of GFP-Cnp1 in all strains; sheep anti-GFP (from K. Hardwick), mouse anti-α-tubulin loading control (from K. Gull).

### Mass Spectrometry Analysis

An LTQ-Orbitrap mass spectrometer (ThermoElectron) was coupled online to an Agilent 1100 binary nanopump and an HTC PAL autosampler (CTC). To prepare an analytical column with a self-assembled particle frit (Ishihama et al., 2002) C18 material (ReproSil-Pur C18-AQ 3 mm; Dr. Maisch, GmbH) was packed into a spray emitter (75 μm ID, 8 μm opening, 70 mm length; New Objectives) using an air-pressure pump (Proxeon). Mobile phase A consisted of water, 5% acetonitrile, and 0.5% acetic acid; mobile phase B consisted of acetonitrile and 0.5% acetic acid. The gradient went from 0% to 20% buffer B in 75 min and then to 80% B in 13 min at 300 nl/min flow. The six most intense peaks of the MS scan were selected in the ion trap for MS^2^, (normal scan, wideband activation, filling 5 × 10^5^ ions for MS scan, 10^4^ ions for MS^2^, maximum fill time 100 ms, dynamic exclusion for 180 s). Raw files were processed using DTAsupercharge 0.62 (a kind gift from M. Mann). The generated peak lists were searched against the SGD database (version 11.05.2007) using Mascot 2.0 with the parameters: monoisotopic masses, 8 ppm peptide tolerance, and 0.6 Da MS/MS tolerance, ESI TRAP parameters, fully tryptic specificity, with two missed cleavage sites allowed, and including serine-threonine phosphorylation. The results were parsed through MSQuant (http://msquant.sourceforge.net/), and a cutoff 5 ppm peptide tolerance was applied to the recalibrated list. Peptides with scores 25 and higher were reported and in individual cases manually validated.

## Supplemental Data

Document S1. Twelve Figures

## References

[bib1] Ahmad K., Henikoff S. (2002). Histone H3 variants specify modes of chromatin assembly. Proc. Natl. Acad. Sci. USA.

[bib2] Allshire R.C., Javerzat J.P., Redhead N.J., Cranston G. (1994). Position effect variegation at fission yeast centromeres. Cell.

[bib3] Allshire R.C., Nimmo E.R., Ekwall K., Javerzat J.P., Cranston G. (1995). Mutations derepressing silent centromeric domains in fission yeast disrupt chromosome segregation. Genes Dev..

[bib4] Aravind L., Iyer L.M., Wu C. (2007). Domain architectures of the Scm3p protein provide insights into centromere function and evolution. Cell Cycle.

[bib5] Bhargava M.M., Cramer J.H., Halvorson H.O. (1972). Isolation of high molecular weight DNA from yeast nuclei. Anal. Biochem..

[bib6] Black B.E., Jansen L.E., Maddox P.S., Foltz D.R., Desai A.B., Shah J.V., Cleveland D.W. (2007). Centromere identity maintained by nucleosomes assembled with histone H3 containing the CENP-A targeting domain. Mol. Cell.

[bib7] Blower M.D., Sullivan B.A., Karpen G.H. (2002). Conserved organization of centromeric chromatin in flies and humans. Dev. Cell.

[bib8] Camahort R., Li B., Florens L., Swanson S.K., Washburn M.P., Gerton J.L. (2007). Scm3 is essential to recruit the histone h3 variant cse4 to centromeres and to maintain a functional kinetochore. Mol. Cell.

[bib9] Carroll C.W., Straight A.F. (2006). Centromere formation: from epigenetics to self-assembly. Trends Cell Biol..

[bib10] Castillo A.G., Mellone B.G., Partridge J.F., Richardson W., Hamilton G.L., Allshire R.C., Pidoux A.L. (2007). Plasticity of fission yeast CENP-A chromatin driven by relative levels of histone H3 and H4. PLoS Genet..

[bib11] Chen Y., Baker R.E., Keith K.C., Harris K., Stoler S., Fitzgerald-Hayes M. (2000). The N terminus of the centromere H3-like protein Cse4p performs an essential function distinct from that of the histone fold domain. Mol. Cell. Biol..

[bib12] Cleveland D.W., Mao Y., Sullivan K.F. (2003). Centromeres and kinetochores: from epigenetics to mitotic checkpoint signaling. Cell.

[bib13] Dalal Y., Wang H., Lindsay S., Henikoff S. (2007). Tetrameric structure of centromeric nucleosomes in interphase Drosophila cells. PLoS Biol..

[bib14] Dunleavy E.M., Pidoux A.L., Monet M., Bonilla C., Richardson W., Hamilton G.L., Ekwall K., McLaughlin P.J., Allshire R.C. (2007). A NASP (N1/N2)-related protein, Sim3, binds CENP-A and is required for its deposition at fission yeast centromeres. Mol. Cell.

[bib15] Foltz D.R., Jansen L.E., Black B.E., Bailey A.O., Yates J.R., Cleveland D.W. (2006). The human CENP-A centromeric nucleosome-associated complex. Nat. Cell Biol..

[bib16] Fujita Y., Hayashi T., Kiyomitsu T., Toyoda Y., Kokubu A., Obuse C., Yanagida M. (2007). Priming of centromere for CENP-A recruitment by human hMis18alpha, hMis18beta, and M18BP1. Dev. Cell.

[bib17] Funabiki H., Hagan I., Uzawa S., Yanagida M. (1993). Cell cycle-dependent specific positioning and clustering of centromeres and telomeres in fission yeast. J. Cell Biol..

[bib18] Furuyama T., Dalal Y., Henikoff S. (2006). Chaperone-mediated assembly of centromeric chromatin in vitro. Proc. Natl. Acad. Sci. USA.

[bib19] Hagan I., Yanagida M. (1995). The product of the spindle formation gene sad1+ associates with the fission yeast spindle pole body and is essential for viability. J. Cell Biol..

[bib20] Hayashi T., Fujita Y., Iwasaki O., Adachi Y., Takahashi K., Yanagida M. (2004). Mis16 and Mis18 are required for CENP-A loading and histone deacetylation at centromeres. Cell.

[bib21] Henikoff S. (2008). Nucleosome destabilization in the epigenetic regulation of gene expression. Nat. Rev. Genet..

[bib22] Ishihama Y., Rappsilber J., Andersen J.S., Mann M. (2002). Microcolumns with self-assembled particle frits for proteomics. J. Chromatogr. A..

[bib23] Jansen L.E., Black B.E., Foltz D.R., Cleveland D.W. (2007). Propagation of centromeric chromatin requires exit from mitosis. J. Cell Biol..

[bib24] Karpen G.H., Allshire R.C. (1997). The case for epigenetic effects on centromere identity and function. Trends Genet..

[bib25] Krapp A., Schmidt S., Cano E., Simanis V. (2001). *S. pombe* cdc11p, together with sid4p, provides an anchor for septation initiation network proteins on the spindle pole body. Curr. Biol..

[bib26] Liu X., McLeod I., Anderson S., Yates J.R., He X. (2005). Molecular analysis of kinetochore architecture in fission yeast. EMBO J..

[bib27] Maddox P.S., Hyndman F., Monen J., Oegema K., Desai A. (2007). Functional genomics identifies a Myb domain-containing protein family required for assembly of CENP-A chromatin. J. Cell Biol..

[bib28] Maruyama T., Nakamura T., Hayashi T., Yanagida M. (2006). Histone H2B mutations in inner region affect ubiquitination, centromere function, silencing and chromosome segregation. EMBO J..

[bib29] Mellone B.G., Allshire R.C. (2003). Stretching it: putting the CEN(P-A) in centromere. Curr. Opin. Genet. Dev..

[bib30] Mizuguchi G., Xiao H., Wisniewski J., Smith M.M., Wu C. (2007). Nonhistone Scm3 and histones CenH3-H4 assemble the core of centromere-specific nucleosomes. Cell.

[bib31] Moreno S., Klar A., Nurse P. (1991). Molecular genetic analysis of fission yeast Schizosaccharomyces pombe. Methods Enzymol..

[bib32] Obuse C., Iwasaki O., Kiyomitsu T., Goshima G., Toyoda Y., Yanagida M. (2004). A conserved Mis12 centromere complex is linked to heterochromatic HP1 and outer kinetochore protein Zwint-1. Nat. Cell Biol..

[bib33] Obuse C., Yang H., Nozaki N., Goto S., Okazaki T., Yoda K. (2004). Proteomics analysis of the centromere complex from HeLa interphase cells: UV-damaged DNA binding protein 1 (DDB-1) is a component of the CEN-complex, while BMI-1 is transiently co-localized with the centromeric region in interphase. Genes Cells.

[bib34] Oeffinger M., Wei K.E., Rogers R., DeGrasse J.A., Chait B.T., Aitchison J.D., Rout M.P. (2007). Comprehensive analysis of diverse ribonucleoprotein complexes. Nat. Methods.

[bib35] Okada M., Cheeseman I.M., Hori T., Okawa K., McLeod I.X., Yates J.R., Desai A., Fukagawa T. (2006). The CENP-H-I complex is required for the efficient incorporation of newly synthesized CENP-A into centromeres. Nat. Cell Biol..

[bib36] Partridge J.F., Borgstrom B., Allshire R.C. (2000). Distinct protein interaction domains and protein spreading in a complex centromere. Genes Dev..

[bib37] Pidoux A.L., Richardson W., Allshire R.C. (2003). Sim4: a novel fission yeast kinetochore protein required for centromeric silencing and chromosome segregation. J. Cell Biol..

[bib38] Pidoux A.L., Allshire R.C. (2004). Kinetochore and heterochromatin domains of the fission yeast centromere. Chromosome Res..

[bib39] Rappsilber J., Ishihama Y., Mann M. (2003). Stop and go extraction tips for matrix-assisted laser desorption/ionization, nanoelectrospray, and LC/MS sample pretreatment in proteomics. Anal. Chem..

[bib40] Rappsilber J., Mann M., Ishihama Y. (2007). Protocol for micro-purification, enrichment, pre-fractionation and storage of peptides for proteomics using StageTips. Nat. Protocols.

[bib41] Schuh M., Lehner C.F., Heidmann S. (2007). Incorporation of *Drosophila* CID/CENP-A and CENP-C into centromeres during early embryonic anaphase. Curr. Biol..

[bib42] Shelby R.D., Monier K., Sullivan K.F. (2000). Chromatin assembly at kinetochores is uncoupled from DNA replication. J. Cell Biol..

[bib43] Song J.S., Liu X., Liu X.S., He X. (2008). A high-resolution map of nucleosome positioning on a fission yeast centromere. Genome Res..

[bib44] Stoler S., Rogers K., Weitze S., Morey L., Fitzgerald-Hayes M., Baker R.E. (2007). Scm3, an essential Saccharomyces cerevisiae centromere protein required for G2/M progression and Cse4 localization. Proc. Natl. Acad. Sci. USA.

[bib45] Sullivan K.F. (2001). A solid foundation: functional specialization of centromeric chromatin. Curr. Opin. Genet. Dev..

[bib46] Sullivan K.F., Hechenberger M., Masri K. (1994). Human CENP-A contains a histone H3 related histone fold domain that is required for targeting to the centromere. J. Cell Biol..

[bib47] Sullivan B., Karpen G. (2001). Centromere identity in Drosophila is not determined in vivo by replication timing. J. Cell Biol..

[bib48] Sullivan B.A., Blower M.D., Karpen G.H. (2001). Determining centromere identity: cyclical stories and forking paths. Nat. Rev. Genet..

[bib49] Takahashi K., Chen E.S., Yanagida M. (2000). Requirement of Mis6 centromere connector for localizing a CENP-A-like protein in fission yeast. Science.

[bib50] Takahashi K., Takayama Y., Masuda F., Kobayashi Y., Saitoh S. (2005). Two distinct pathways responsible for the loading of CENP-A to centromeres in the fission yeast cell cycle. Philos. Trans. R. Soc. Lond. B Biol. Sci..

[bib51] Takayama Y., Sato H., Saitoh S., Ogiyama Y., Masuda F., Takahashi K. (2008). Biphasic incorporation of centromeric histone CENP-A in fission yeast. Mol. Biol. Cell.

[bib52] Westermann S., Drubin D.G., Barnes G. (2007). Structures and functions of yeast kinetochore complexes. Annu. Rev. Biochem..

[bib53] Williams S.K., Tyler J.K. (2007). Transcriptional regulation by chromatin disassembly and reassembly. Curr. Opin. Genet. Dev..

[bib54] Williams J.S., Hayashi T., Yanagida M., Russell P. (2009). Fission yeast Scm3 mediates stable assembly of Cnp1/CENP-A into centromeric chromatin. Mol. Cell.

[bib55] Zofall M., Grewal S.I. (2007). HULC, a histone H2B ubiquitinating complex, modulates heterochromatin independent of histone methylation in fission yeast. J. Biol. Chem..

