# Supplementary material for: Fission Yeast Scm3: A CENP-A Receptor Required for Integrity of Subkinetochore Chromatin
Source: Mol Cell. 2009 Feb 13;33(3-3):299–311. doi: 10.1016/j.molcel.2009.01.019 (PMC2697330; doi:10.1016/j.molcel.2009.01.019)
Supplement: Document S1. Twelve Figures [file mmc1.pdf]

Molecular Cell, Volume *33*

Supplemental Data

Fission Yeast Scm3: A CENP-A Receptor Required for Integrity of Subkinetochore Chromatin

Alison L. Pidoux, Eun Shik Choi, Johanna K. R. Abbott, Xingkun Liu, Alexander Kagansky, Araceli G. Castillo, Georgina L. Hamilton, William Richardson, Juri Rappsilber, Xiangwei He, and Robin C. Allshire

Figure S1. *sim1* mutants have altered central domain chromatin.

Wild-type or *sim1* mutant strains were incubated at 25°C (permissive temperature) or 36°C (restrictive) for 6 hours. Permeabilised cells were treated with increasing concentrations of Micrococcal nuclease (left to right) to partially digest chromatin. Extracted DNA was subjected to electrophoresis in a 1.2% agarose gel (Ethidium bromide), revealing a typical nucleosomal ladder. Southern blot was hybridised with a central core probe (*cnt*) to reveal the unusual smear pattern of sub-kinetochore chromatin. Wild-type is identical to that in Pidoux et al. (2003).

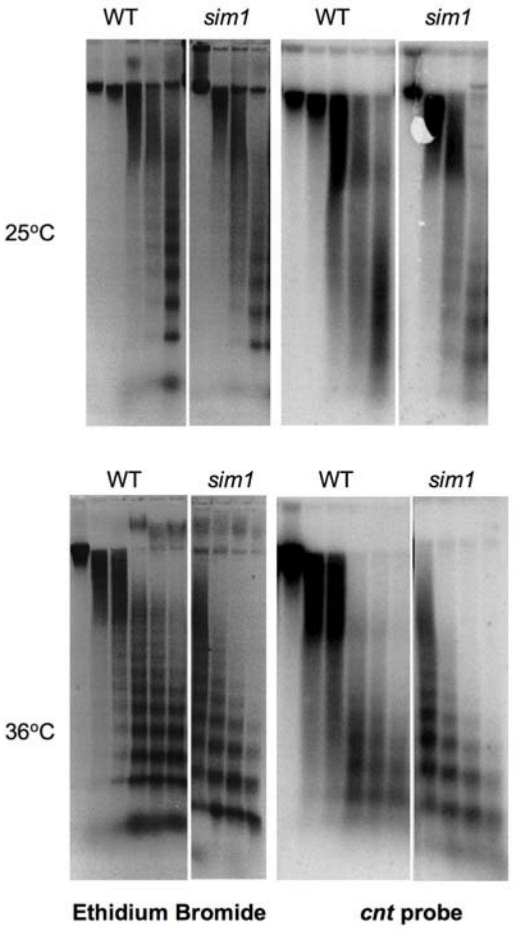

Figure S2. Frequency of segregation defects in wild type and *sim1-15* mutant

Cells were treated as in Fig. 1B and cells examined for frequency of hypercondensed chromatin (indicative of early mitotic block), lagging chromosomes, or uneven segregation.

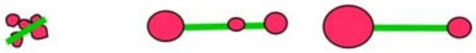

Strain	Hyper-condensed DNA	Lagging chromosomes	Unequal segregation of DNA
**Wild type 25°C**	**0.0%** (0/108)	**0.0%** (0/108)	**0.0%** (0/108)
**Wild type 36°C**	**1.5%** (3/195)	**0.5%** (1/195)	**0.0%** (0/195)
sim1-15 **25°C**	**1.6%** (2/124)	**3.2%** (4/124)	**3.2%** (4/124)
sim1-15 **36°C**	**13.8%** (27/196)	**8.2%** (16/196)	**10.7%** (21/196)

Figure S3. *scm3*$^{+}$ is an essential gene.

The *scm3*$^{+}$ ORF was replaced by the *ura4*$^{+}$ gene in a diploid strain. The heterozygous diploid was induced to sporulate and tetrads dissected (top). Viable colonies from these and additional tetrads were confirmed to be auxotrophic for uracil, indicating that *scm3Δ::ura4*$^{+}$ cells are inviable.

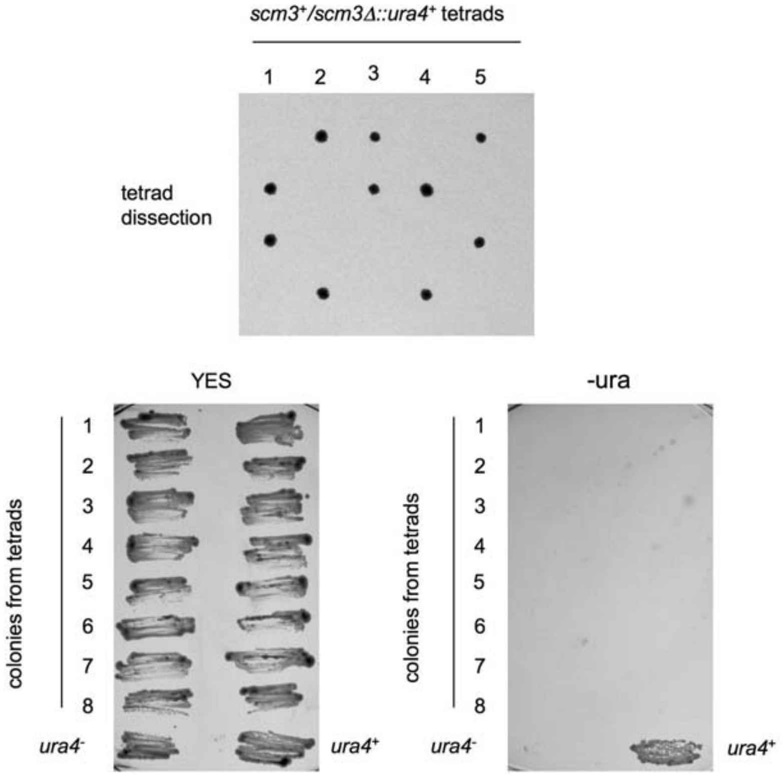

Figure S4. Scm3^{Sp} dissociates from centromeres at the beginning of mitosis, whereas CENP-A^{Cnp1} remains associated.

Top: Cells fixed and stained for immunofluorescence with rabbit anti-Scm3 (red) antibodies and sheep anti-Cnp1/CENP-A^{Cnp1} antisera (green).
Bottom: Cells expressing Scm3-GFP were fixed and stained for immunofluorescence with rabbit anti-GFP antibodies (green) and sheep Cnp1/CENP-A^{Cnp1} antisera (red).
In both cases Scm3 disappears as cells enter mitosis and centromeres decluster and separate in early mitosis. Cells were stained with DAPI (blue). Scale bars, 5 μm.

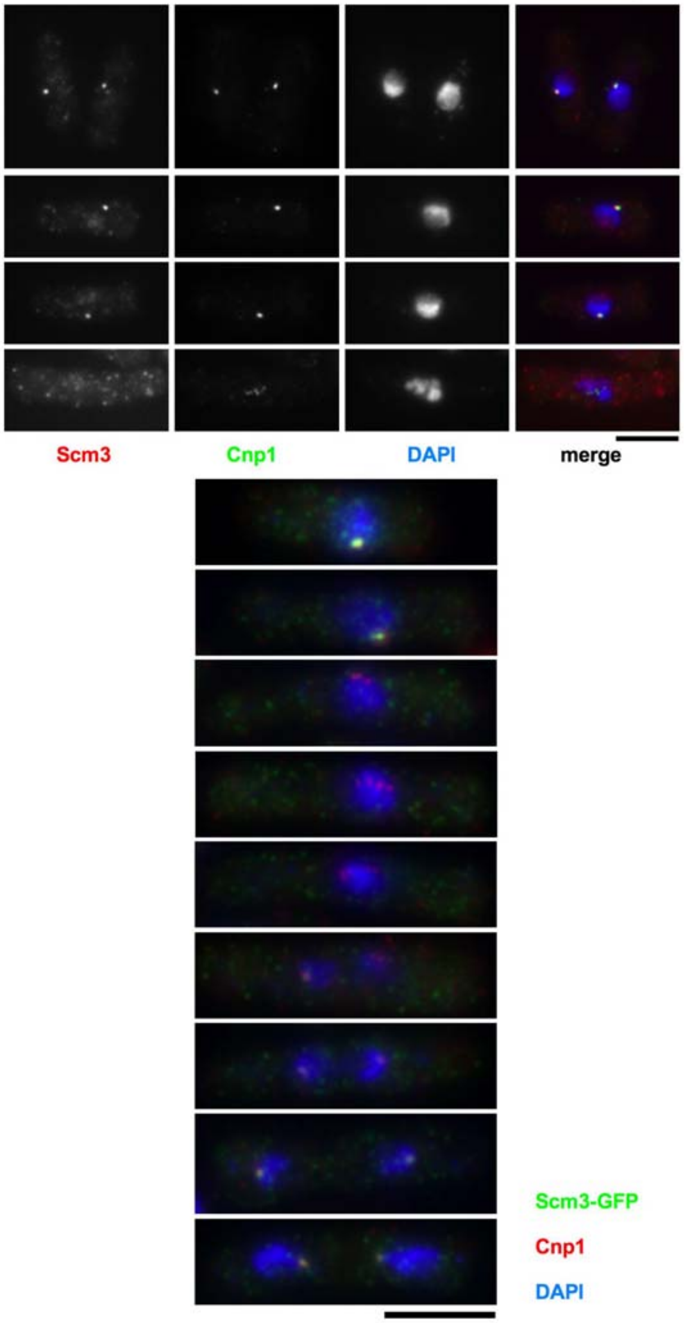

Figure S5. CENP-A^{Cnp1} and Scm3-GFP are enriched over the entire central core domain.

ChIP was performed with anti-Cnp1/CENP-A^{Cnp1} control locus (*fbp1*) and anti-GFP (Scm3-GFP) antibodies and analysed with primer pairs (1-19) space that amplify fragments as indicated on the diagram of the fission yeast *cen1*: central core (*cnt1*), innermost repeats (imr1L and imr1R) and outer repeats (otr/dg). Small black arrows indicate tRNA genes, the left and right pair of tRNA gnes are located at the transition between sub-kinetochore (central domain) chromatin and heterochromatin. Enrichment relative to euchromatic control locus (*fbp1*) is indicated. Primer pair 1 is within heterochromatin outside the sub-kinetochore chromatin domain and provide a negative control. For Cnp1, and Scm3-GFP ChIP the ratio of the PCR products cen:fbp1 was normalized with respect to the cen:fbp1 ratio observed in the corresponding Total extract.

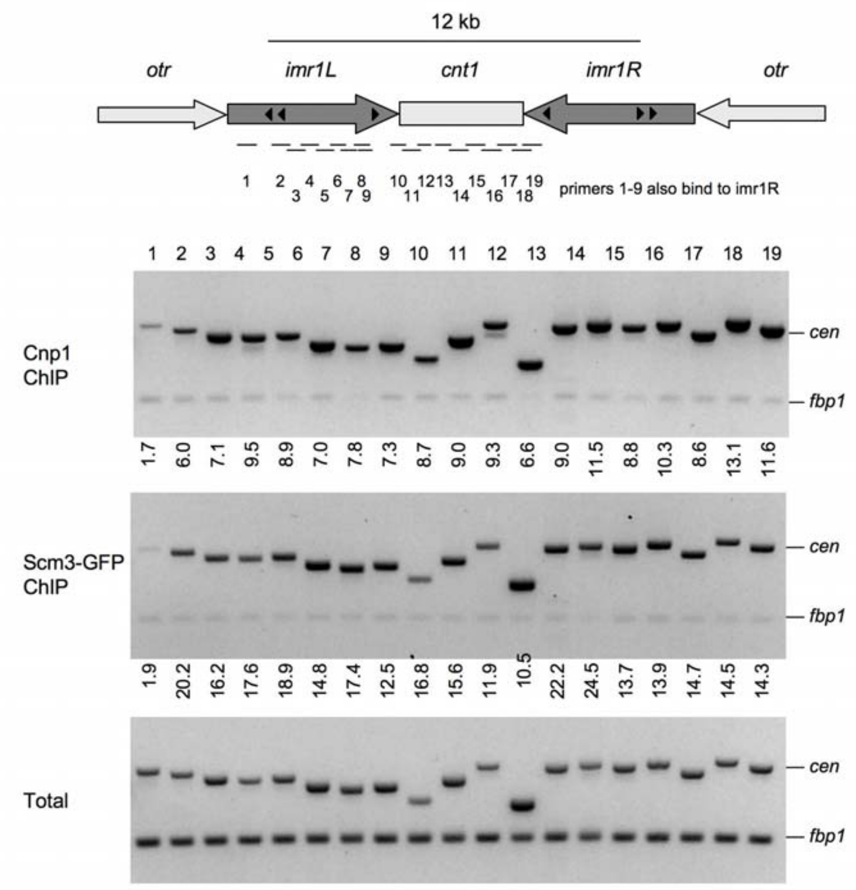

Figure S6. Scm3-GFP remains localised in *cnp1-1* cells even when CENP-A^{Cnp1} is undetectable at centromeres.

Wild type (shown in Fig. 3B) and *cnp1-1* strains expressing Scm3-GFP were shifted to 36°C for 6 hours, fixed and processed for immunofluorescence using anti-GFP (Scm3-GFP: green), and anti-Cnp1/CENP-A^{Cnp1} antibodies (red), and DAPI (blue). Identical exposures and processing were performed to ensure that wild-type and mutant images are comparable. Representative images are presented. Scale bar 5 μm.

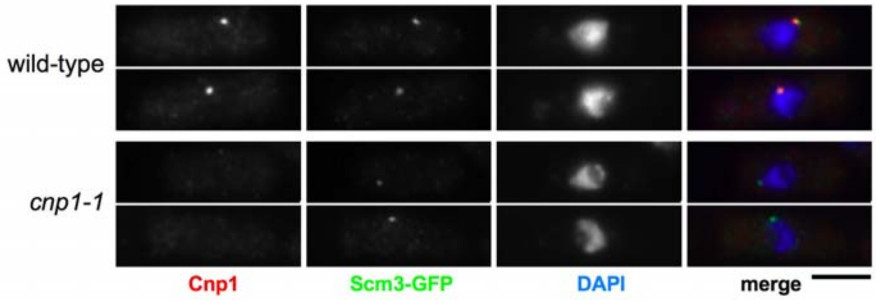

Figure S7. H2B-FLAG ChIP across centromere 1 shows a similar profile to that of H3

ChIP using anti-FLAG antibodies to assess enrichment of H2B-FLAG in the central domain (*cnt*) relative to euchromatic control region (*fbp1*) in a strain expressing H2B-FLAG (only source of H2B). Cnp1/CENP-A^{Cnp1} and H3 ChIPs were also performed for comparison. Primer pairs (1-19) that amplify fragments as indicated on the diagram of the fission yeast *cen1* (see Fig. S5). Graphs: Enrichment of centromere regions relative to euchromatic control locus (*fbp1*) is indicated. For Cnp1, H2B-FLAG and H3 ChIP the ratio of the PCR products cen:fbp1 was normalized with respect to the cen:fbp1 ratio observed in the corresponding Total extract.

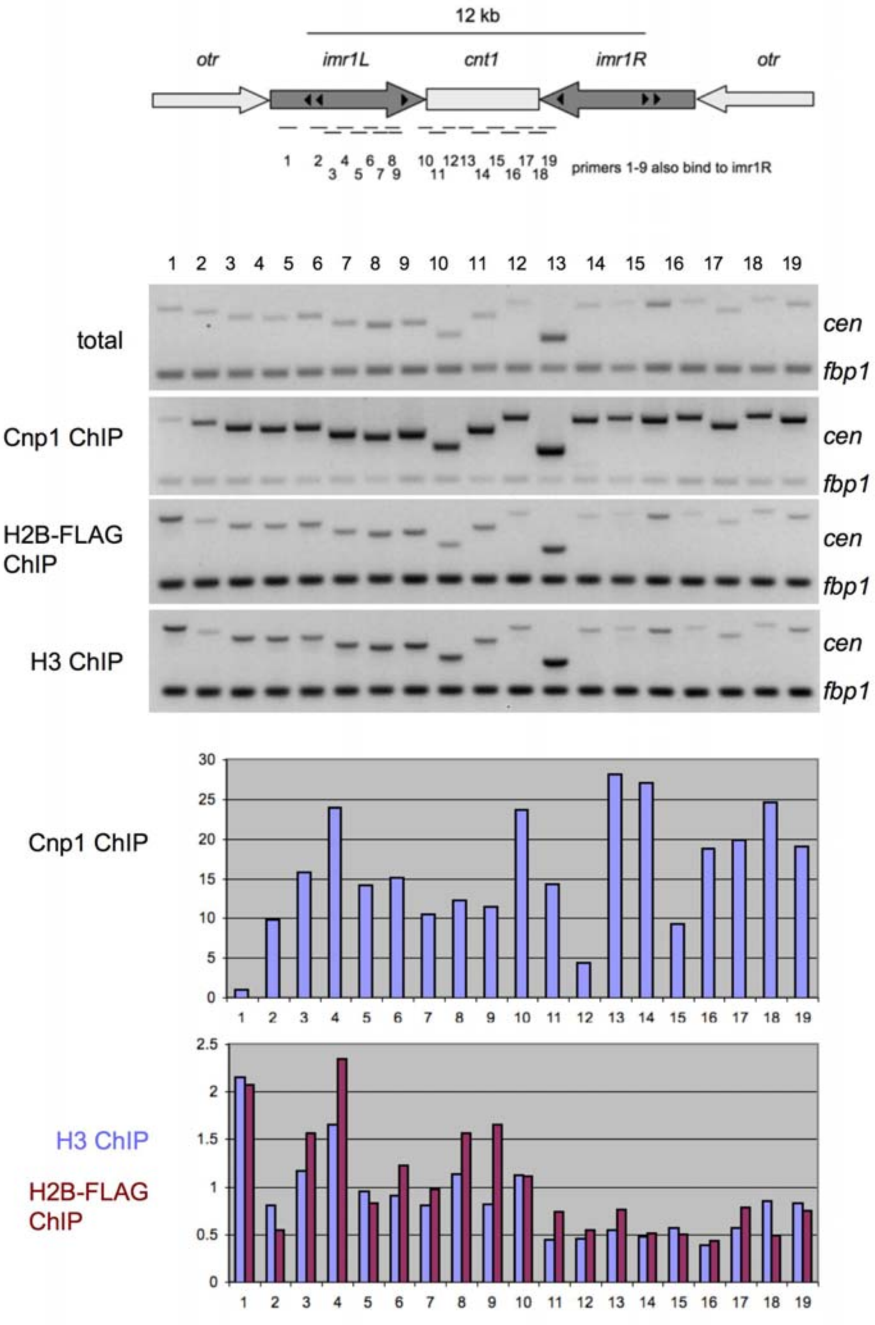

Figure S8. CENP-A^{Cnp1}-TAP and Histone H4 are released from chromatin by MNase digestion, Scm3-TAP remains in the pellet.

A. Nuclear Chromatin was digested to oligo-nucleosomal particles (mono-, di-, and tri-nucleosomes are most prominent) which were released into the supernatant. DNA was purified from chromatin before or after 7 minutes MNase digestion and separated by agarose electrophoresis.
B. Chromatin digested for 7 minutes with MNase was centrifuged, and proteins were extracted from a portion of the digestion mixture (Total), the supernatant (Sup.) and Pellet. Protein were also extracted from untreated chromatin (No MNase). Scm3-TAP, CENP-A^{Cnp1}-TAP and histone H4 were detected by Western analyses.
Asterisk indicates non-specific band.

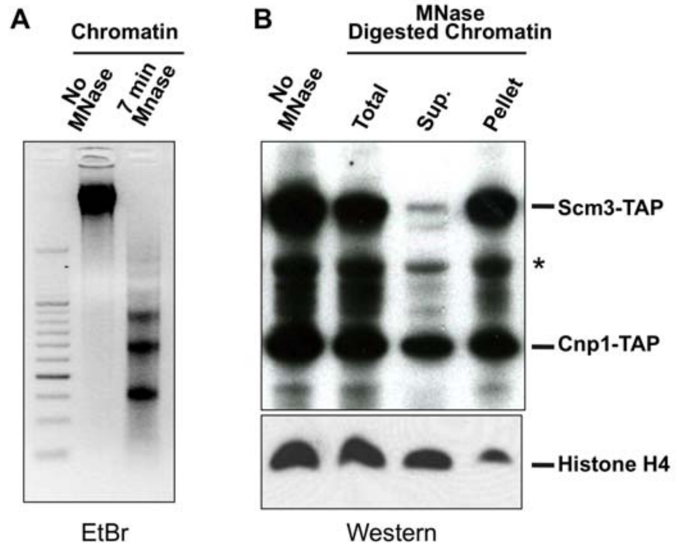

Figure S9. Scm3-GFP remains localised in mutants defective in Cnp3$^{CENP\text{-}C}$, Sim3 or Mis12.

A. Immunolocalisation: Wild-type, *cnp3Δ* and *sim3Δ* cells expressing Scm3-GFP were grown at 36°C (permissive temperature), fixed and stained for immunofluorescence with rabbit anti-GFP antibodies (green), and sheep anti-Cnp1/CENP-A^{Cnp1} antisera (red). In a separate experiment, wild-type and *mis12-537* cells were shifted to restrictive temperature (36°C) for 6 hours before fixation and processing as above. Scale bar, 5 μm.
B. ChIP: ChIP for Scm3-GFP and CENP-A^{Cnp1} was also performed was also performed on cultures treated similarly to above, as described in Fig. 3B.

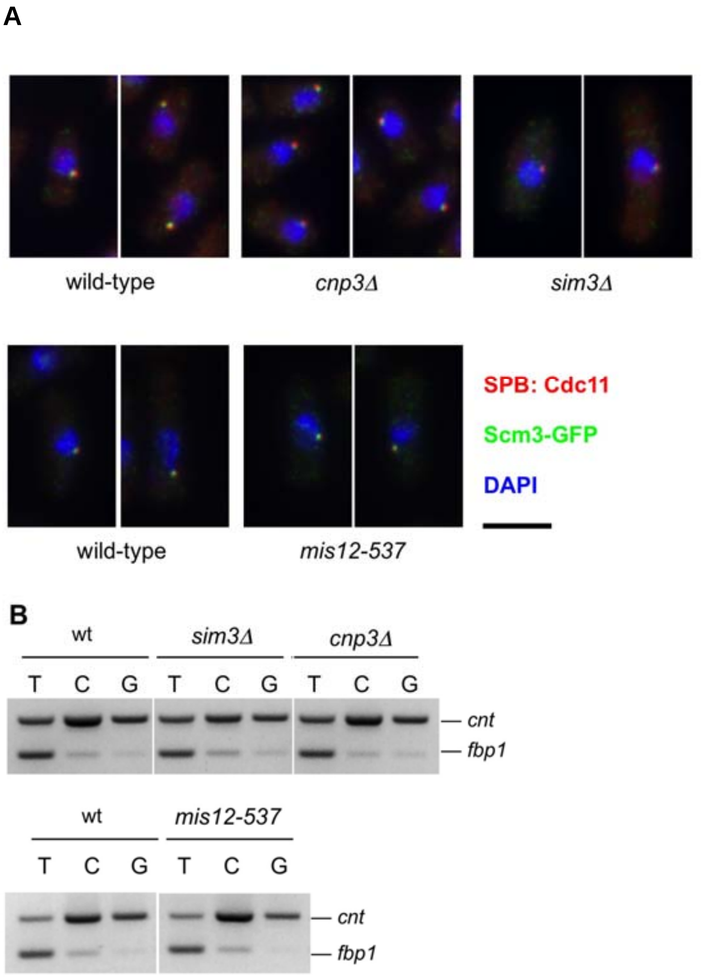

Figure S10. Interactions between CENP-A^{Cnp1} and Scm3

A Scm3-13myc is immunoprecipitated with anti-Cnp1 antiserum. Western blot with rabbit anti-myc antibody (A14; Santa Cruz). U, untagged; WCE, whole cell extract; IP, immunoprecipitation with sheep anti-Cnp1 anti-serum.
B Deletion of the gene encoding Sim3, a CENP-A^{Cnp1} escort, disrupts the interaction between CENP-A^{Cnp1} and Scm3^{Sp}. Co-affinity purification was performed as in Figure 7 on *sim3*$^{+}$ or *sim3Δ* cells with expressing Scm3-CTAP at endogenous levels and overexpressing CENP-A^{Cnp1}-GFP. Soluble cell extracts were subject to affinity purification using IgG-beads. Input samples (I) and bead-bound samples (B) were subject to Western blotting to detect Scm3-CTAP and CENP-A^{Cnp1}-GFP.

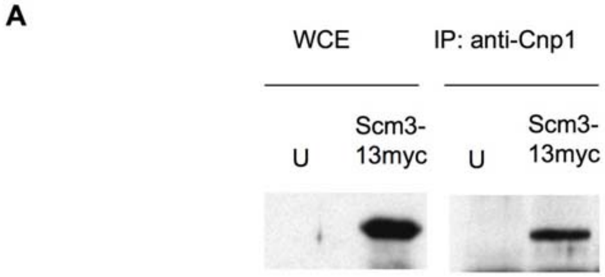

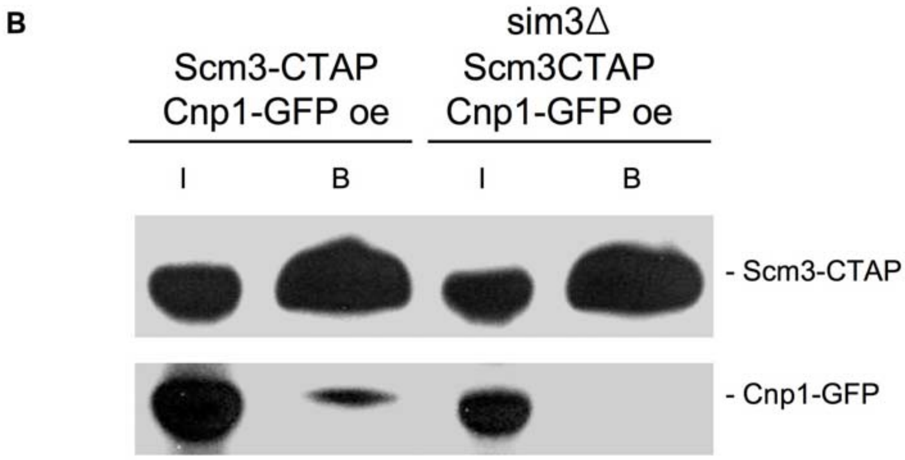

Figure S11. Scm3 function is required for proper incorporation of newly-synthesized GFP-Cnp1

A-D Expression of newly synthesized GFP-Cnp1 from the *inv1* promoter was induced by growing cells in medium containing 4% sucrose (Experimental procedures). Cells were grown at restrictive temperature of 32°C for a total of 6.5 h (induced time-point 2). Fluorescence images of fixed cells are presented. Images were obtained with identical exposure times, scaling and processing. Two wild-type cultures (A&B) and two *scm3-139* cultures (C&D) were analysed.
E Cells from induced time-point 2 in the indicated cultures were categorized by eye as having a bright or faint GFP-Cnp1 spot or having GFP-Cnp1 distributed through the nucleus.
F Western analyses of extracts made from repressed (R) or induced cells (time-points 1, 2 and 3). Sheep anti-GFP was used to assess the level of GFP-Cnp1 and mouse anti-tubulin was used as a loading control.

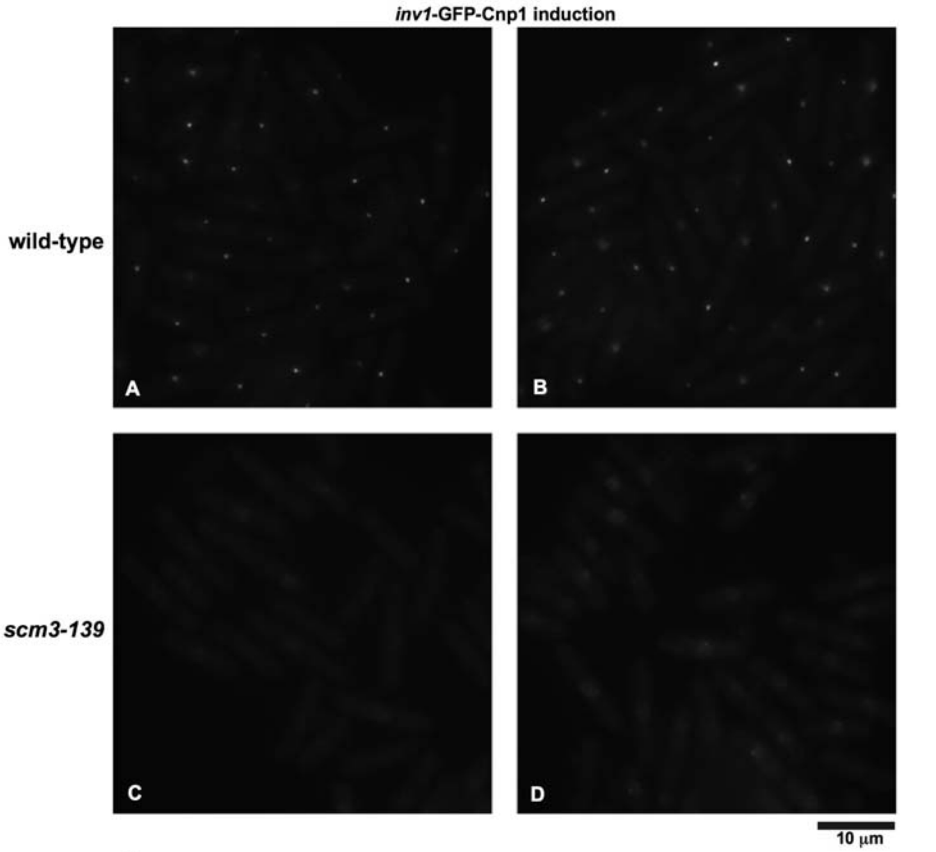

E

	Bright spot	Faint spot	Nucleoplasm	
wild-type A	98%	2%	-	n=100
wild-type B	95%	5%	-	n=100
scm3-139 C	-	1%	5%	n=116
scm3-139 D	-	11%	28%	n=112

F

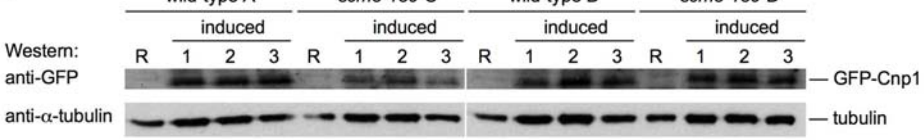

Figure S12. Scm3^{Sp} is phosphorylated at Serine 127

m/z values designated with an asterisk include the mass of phosphate but were observed with neutral loss of 98 Da, indicating the loss of phosphate from this fragment and is consistent with the formation of dehydroalanine from phosphoserine. The phosphorylation is hence found within the part of the peptide covered by the respective fragment. In this way, the position of the modification was narrowed down starting from the N-terminus to VSPVHIEDFQSPQIYK and from the C-terminus to AVSPV, leaving VSPV as the possible region of phosphorylation. Observing VHIEDFQSPQIYK as a non-phosphorylated fragment further narrowed the position of the phosphorylation site to VS. This is fully consistent with S(127) being the site of phosphorylation and excludes the possibility of S(136) being phosphorylated in this peptide.

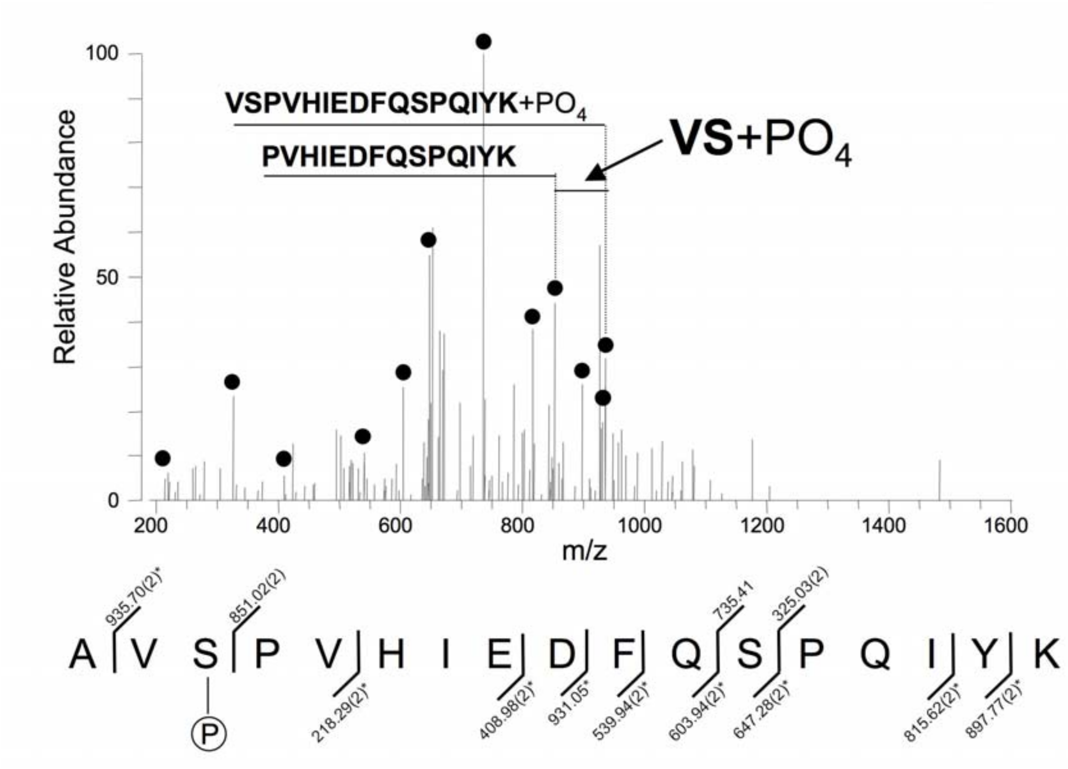

Supplemental Reference

Pidoux, A.L., Richardson, W., and Allshire, R.C. (2003). Sim4: a novel fission yeast kinetochore protein required for centromeric silencing and chromosome segregation. J Cell Biol *161*, 295-307.
